# Faunistic Catalog of the Caddisflies (Insecta: Trichoptera) of Parque Nacional do Itatiaia and its Surroundings in Southeastern Brazil

**DOI:** 10.1673/031.012.2501

**Published:** 2012-02-15

**Authors:** Leandro Lourenço Dumas, Jorge Luiz Nessimian

**Affiliations:** Departamento de Zoologia, Instituto de Biologia, Universidade Federal do Rio de Janeiro, Caixa Postal 68044, Cidade Universitária, 21941-971, Rio de Janeiro, RJ, Brazil

**Keywords:** *Antarctoecia*, Atlantic Forest, biogeography, *Neoatriplectides*, Neotropics

## Abstract

The Atlantic Forest is considered one of the world's biological diversity hotspots, and is increasingly threatened by the rapid destruction and fragmentation of its natural areas. The caddisflies (Trichoptera) of Itatiaia massif, an Atlantic Forest highland area, are inventoried and cataloged here. The catalog is based on examination of bibliographies, field work on many localities of Itatiaia massif (including Parque Nacional do Itatiaia — PNI), and the entomological collection Professor José Alfredo Pinheiro Dutra (DZRJ), Universidade Federal do Rio de Janeiro. A total of 92 species are recorded, representing about 17% of the known Brazilian Trichoptera fauna. Leptoceridae, Hydropsychidae, and Philopotamidae are the families most represented. The high species richness, as well as the remarkable patterns of species distribution, may be related to the characteristics of Mantiqueira mountain range.

## Introduction

Caddisflies (Trichoptera) comprise more than 13,500 extant species described from all faunal regions, arranged in about 610 genera and 47 families (Morse 2011). However, Schmid (1984) claimed that world fauna may contain approximately 50,000 species, which leads to the conclusion that only about 25% of world species of caddisflies have been described. Even limited to the diversity currently known, Trichoptera constitutes the 7th-largest insect order and the most diverse among orders of primary aquatic insects (Paprocki et al. 2004; Holzenthal et al. 2007).

The Neotropical Region is divided into two distinct faunal subregions: the Chilean subregion (southern Chile and adjacent Argentina) and the Brazilian subregion (southern Mexico, Central America, Antilles, and remnant South America) (Flint 1976). de Moor and Ivanov (2008) proposed an alternative biogeographic pattern for Trichoptera distribution, considering 12 biogeographical regions. According to them, the Neotropical region *sensu* Wallace (1876) is divided into Patagonian and Neotropical regions, corresponding respectively to the Chilean and Brazilian subregions, as determined by Flint (1976). Chilean fauna is highly endemic and closely related to the fauna of the Australian region. Furthermore, the northern Andes, the Amazon basin, and the mountains of southern and southeastern Brazil can be considered areas with great concentrations of endemic species and with high numbers of non-endemic species (Flint et al. 1999).

There are about 2,200 species described from the Neotropical region, where diversity and distribution of Trichoptera are little-known (Flint et al. 1999). In Brazil, a recent inventory of the fauna reported 378 species for the country (Paprocki et al. 2004). Currently, this number has increased significantly to approximately 550 species, distributed in 70 genera and 16 families (Santos et al. 2011), indicating that the diversity of the order in Brazil is underestimated. In such case, there are many new species remaining to be discovered and described (Blahnik et al. 2004). There are over 300 new species deposited in Brazilian and in foreign entomological collections waiting to be described (Calor 2009). Besides that, many species are only known from their type-localities (Dumas et al. 2010).

The order Trichoptera is divided into three suborders: Annulipalpia (net-spinning or fixed-retreat makers), Integripalpia (portable-case makers), and Spicipalpia (Hydrobiosidae and Rhyacophylidae (free-living), Glossosomatidae (saddle-case makers), and Hydroptilidae (purse-case makers)) (Holzenthal et al. 2007). However, Spicipalpia is not monophyletic in either morphological or molecular phylogenetic analyses (Morse 1997; Kjer et al. 2001, 2002). Some phylogenetic works recognize to a fourth suborder called Protomeropina, composed of fossil families from the Permian. This suborder is sometimes considered part of the ancestral Amphiesmenoptera lineage (de Moor and Ivanov 2008; Calor 2009).

Immature caddisflies stages are exclusively aquatic, being important in aquatic assemblages. Larvae are important components of energy flow and nutrient dynamics in freshwater environments (Resh and Rosenberg 1984). Trichoptera larvae are capable of spinning silk from modified salivary glands. Silk is used in many ways by caddis larvae to construct portable cases, fixed retreats, shelters, and capture nets, and probably is an asset in their ecological and taxonomic diversification (Wiggins 1996). Immature caddisflies can be found in all types of freshwater environments, being especially diverse in running waters like rivers and streams. Furthermore, larvae of Trichoptera have distinct responses to pollution and other environmental impacts. For this reason, caddisflies are widely used in water quality monitoring programs (Morse 1997; Paprocki et al. 2004). Adult caddisflies resemble small moths, generally drab in color, and are found in riparian and shoreline vegetation (Angrisano 1995a; Holzenthal et al. 2007). In contrast to larvae, ecology and behavior of adult Trichoptera are poorly known (Flint et al. 1999).

The Brazilian Atlantic Forest is among the five most important biodiversity hotspots in the world. Less than 8% of the original forest now remains, and it occurs mostly in isolated topographically remnants scattered throughout a landscape dominated by agricultural uses and urbanization. Despite these disturbances, the Atlantic Forest is still extremely rich in biodiversity, sheltering a significant proportion of the Brazilian fauna and flora with high levels of endemism (Joly and Bicudo 1998; Myers et al. 2000). The Itatiaia massif, on which the Parque Nacional do Itatiaia (PNI) exists, is among one of the most important protected areas of Atlantic Forest due to different forest formations with well-defined climatic and vegetation bands (Ururahy et al. 1983). Trichoptera species recorded from the Itatiaia massif were derived from isolated species descriptions and general checklists for southeastern Brazil. However, a comprehensive checklist was not available for the Itatiaia massif, highlighting the gaps in our knowledge of this group in the area. Furthermore, collection events were concentrated in the lower portion of PNI. Therefore, herein we present a catalog aiming to update the list of caddisfly species found in the Itatiaia massif. This catalog is based on recently collected specimens, specimens previously deposited in the Coleção Entomológica Prof. José Alfredo Pinheiro Dutra, UFRJ, Brazil (DZRJ), and literature data until 2011, providing a taxonomic overview of the Trichoptera species known to occur in the Itatiaia massif. New Brazilian state records and distribution data are also given here.

## Materials and Methods

### Study area

The Itatiaia massif is situated in Mantiqueira mountain range, an extensive area of highlands in southeastern Brazil. The massif is located on the border of three Brazilian states: Minas Gerais (Alagoa, Bocaina de Minas and Itamonte municipalities), Rio de Janeiro (Itatiaia and Resende municipalities), and São Paulo (a small portion of Queluz municipality).

The highlands of Itatiaia massif are a Pre-Cambrian outcrop of metamorphic nephelinesyenite rocks (IBDF 1982). One of the most important Brazilian geological areas, the massif possesses the seventh-highest mountain of the country - the Itatiaiaçu -which stands at 2,787 m, located in Agulhas Negras complex. Other important peaks, such as Pedra do Couto (2,682 MASL) and Prateleiras (2,515 MASL) also belong to the massif (Magro 1999).

**Figures 1–4.**
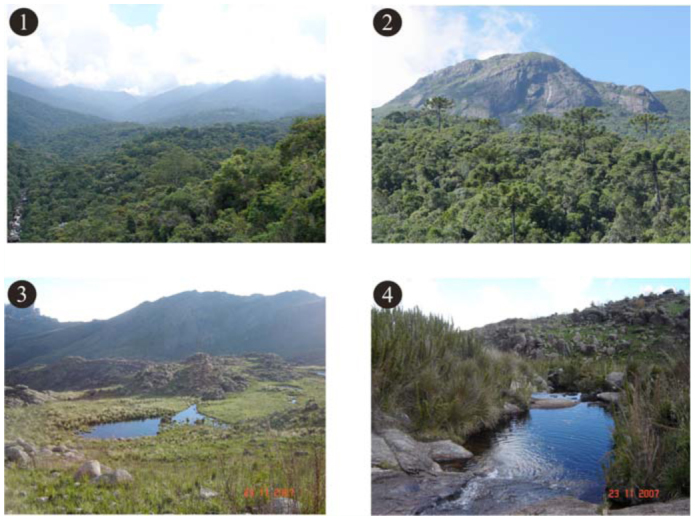
(1) Montane forest at lower portion of PNI; (2) Upper montane forest, with some Araucaria trees; (3) Grassland at Itatiaia plateau, PNI; (4) High-altitude grassland near Agulhas Negras peak, PNI. High quality figures are available online.

Itatiaia massif has four vegetation types that follow an altitudinal gradient: lower montane forest (from 400 to 499 MASL) montane forest (from 500 to 1,499 MASL) (Figure 1), upper montane forest (from 1,500 to 1,999 MASL) (Figure 2), and high-altitude grasslands (more than 2,000 MASL) (Figures 3 and 4) (Ururahy et al. 1983). According to The Conservation International of Brazil (2000), this region is characterized as a nucleus of the Atlantic Forest Biosphere Reserve, one of the biggest conservation units in the world.

The climate is mesothermic, markedly seasonal, with cold and dry winters and very wet summers (Cwa according Köppen's classification). Mean annual temperature is about 14 °C, with lower temperatures falling below –10 °C during the rigorous winter. Annual rainfall is about 2,400 mm, concentrated in the summer months (Ribeiro et al. 2007). At the end of April and beginning of October the rainfalls become uncommon, which causes a relatively dry winter (Barros 2003).

Itatiaia massif is inserted between the Rio Picu gorge (MG) and Mauá (RJ), having Paraíba do Sul drainage basin on the south (Rio de Janeiro State) and Rio Grande drainage basin on the north (Minas Gerais State). The three main rivers of massif which contribute to the Rio Paraíba do Sul basin are: Rio Preto, that drains the northeastern area; Rio Campo Belo, that flows at southeastern portion; and Rio do Salto, located at southwestern section. Rio Capivari (tributary of Rio Verde) and Rio Aiuruoca (tributary of Rio Turvo) are the main rivers that form Rio Grande basin, on northwestern portion of the massif (IBDF 1982). Rivers and streams of Itatiaia massif have regular discharges during the winter, receiving a large amount of water during the summer period. The rivers usually have a tumbling flow, forming rapids along sloping rocky beds, mainly at the south portion (turned to Paraíba do Sul valley), where the topography is more pronounced (Magro 1999). At the high area of the massif, there are highland lakes formed by flowing water of marshes. These may become frozen during the winter (IBAMA 1994).

**Figure 5.**
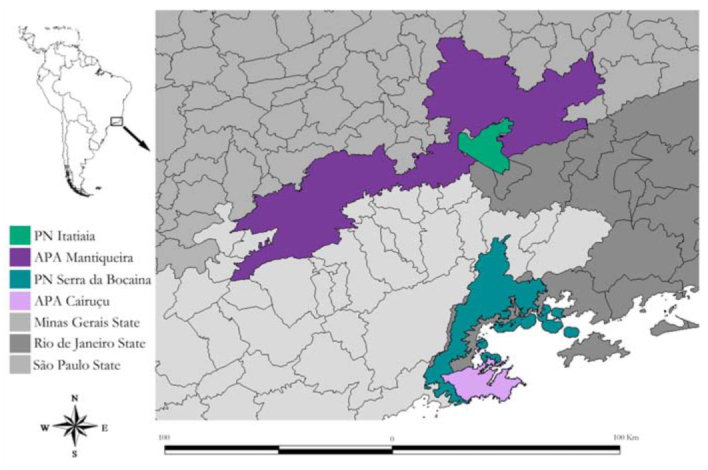
Localization of Parque Nacional do Itatiaia (PNI), southeastern Brazil. High quality figures are available online.

PNI is located on the border between Rio de Janeiro and Minas Gerais states, between 22° 19′ – 22° 45′ S and 44° 15′ – 44° 50′ W. The protected area was established in 1937 and is the oldest national park in Brazil. Currently, PNI comprises an area of 30,000 ha, covering 20% of Itatiaia massif (Barros 2003). The park is surrounded by Área de Proteção Ambiental da Mantiqueira (APA da Mantiqueira) which provides an ecological buffer zone for the park (Figure 5).

**Figures 6–11.**
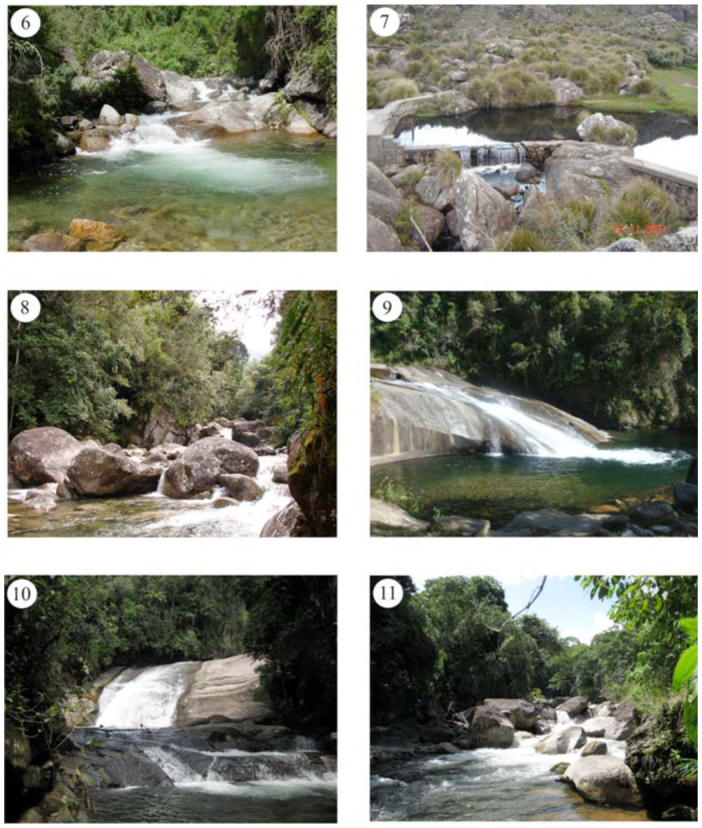
Rivers and streams of Itatiaia massif. (6) Rio Aiuruoca; (7) Rio Campo Belo dam at higher portion of PNI; (8) Rio Campo Belo; (9) Rio Preto, Escorrega do Maromba; (10) Rio das Pedras, Cachoeira de Deus; (11) Rio do Salto. High quality figures are available online.

### Sampling

The catalog of species is based mainly on specimens collected from many localities of the Itatiaia massif between 1990 and 2009. Additional records from previous published articles are also provided here. Sampled area was divided into five major drainage sub-basins (Figures 6–11): (1) Rio Aiuruoca sub-basin (Figure 6), (2) Rio Campo Belo sub-basin (Figures 7 and 8), (3) Rio Preto sub-basin (Figure 9), (4) Rio das Pedras sub-basin (Figure 10), and (5) Rio do Salto sub-basin (Figure 11). In addition, the specimens deposited at Coleção Entomológica Professor José Alfredo Pinheiro Dutra (DZRJ) of the Universidade Federal do Rio de Janeiro were examined. Taxonomic bibliography that includes data of Itatiaia massif is also included here to complement this inventory.

**Figures 12–13.**
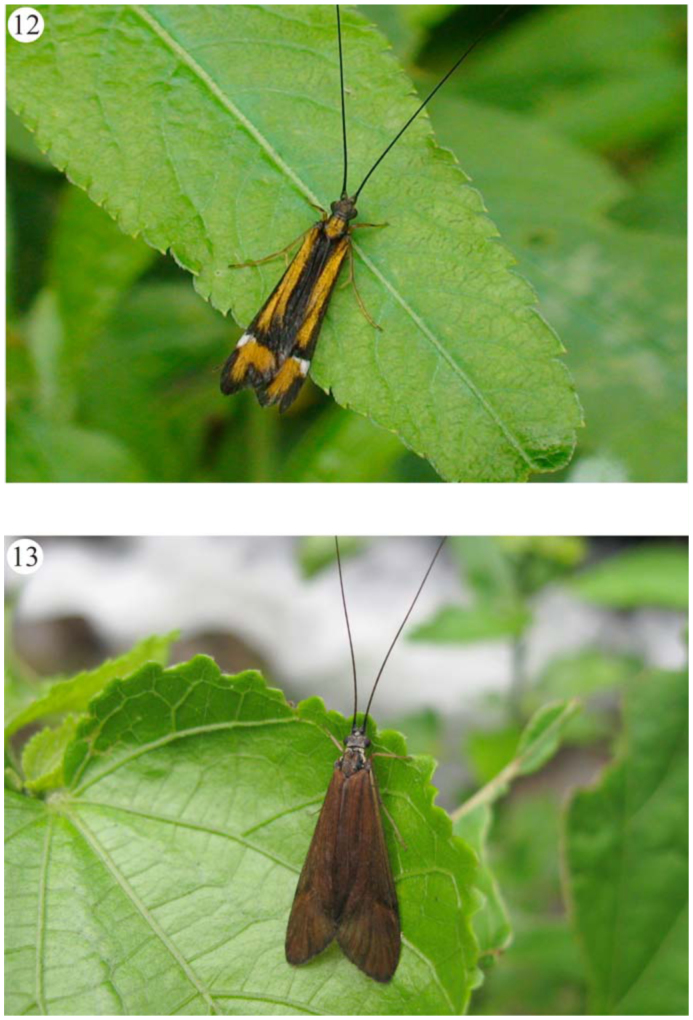
Some caddisflies specimens of Itatiaia massif. (12) Hydropsychidae; (13) *Centromacronema auripenne* (Rambur) (Hydropsychidae). Picture 12 – Marcela Laura Monné. High quality figures are available online.

Larvae and pupae were collected with Surber and Brundin nets (125 µm and 180 µm mesh), sieves, and manually in several kinds of substrate, in rapids, and pools of rivers and streams. The specimens were preserved in 80% ethanol. Adults were collected with light traps (white sheet and Pennsylvania light trap), which were placed near streams and lightened at dusk, remaining switched on during the night. At daytime the adults were collected in activity with entomological nets and aspirators. The specimens were also preserved in 80% ethanol and few ones were pinned.

**Figures 14–15.**
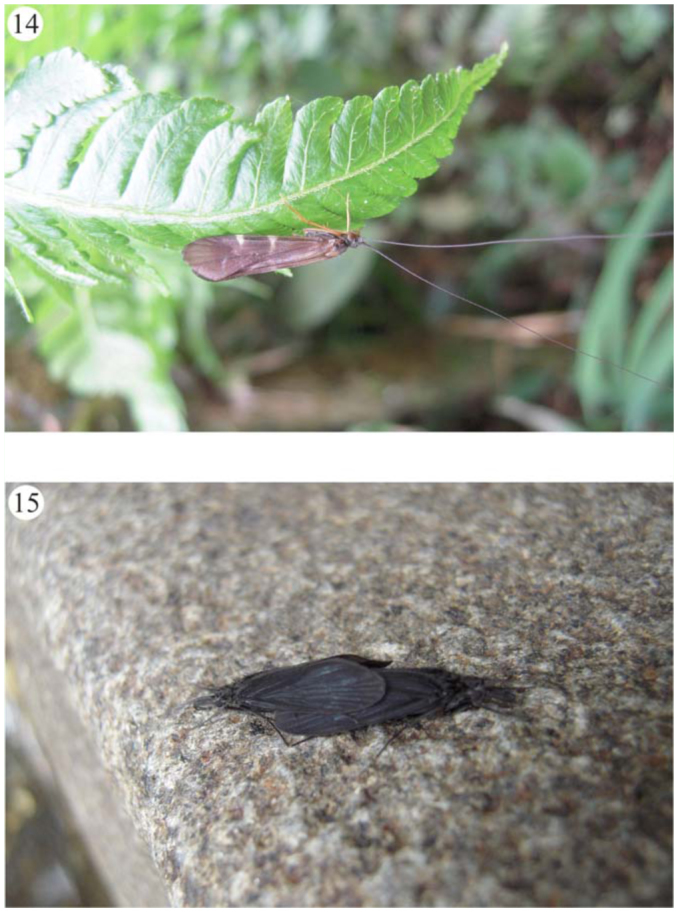
Some caddisflies specimens of Itatiaia massif. (14) *Macrostemum* sp. (Hydropsychidae); (15) *Chimarra* sp., mating (Philopotamidae). Pictures 14 and 15 – Daniela Maeda Takiya. High quality figures are available online.

Immature stages were identified to generical level based on keys by Angrisano (1995a), Wiggins (1996), and Pes et al. (2005). Some larvae were identified to species level according to descriptions given for manuscripts of immature association (Holzenthal 1997; Huamantinco and Nessimian 2004a; Huamantinco et al. 2005; Dumas and Nessimian 2006; Calor and Froehlich 2008; Nessimian and Dumas 2010). Adult identification was based on the morphology of male genitalia. In order to observe the genital structures, the abdomen was removed and cleared in a heated solution of 10% KOH (Beten 1934). The specimens are deposited in Coleção Entomológica Professor José Alfredo Pinheiro Dutra (DZRJ), Departamento de Zoologia, Universidade Federal do Rio de Janeiro, Rio de Janeiro State.

**Figures 16–17.**
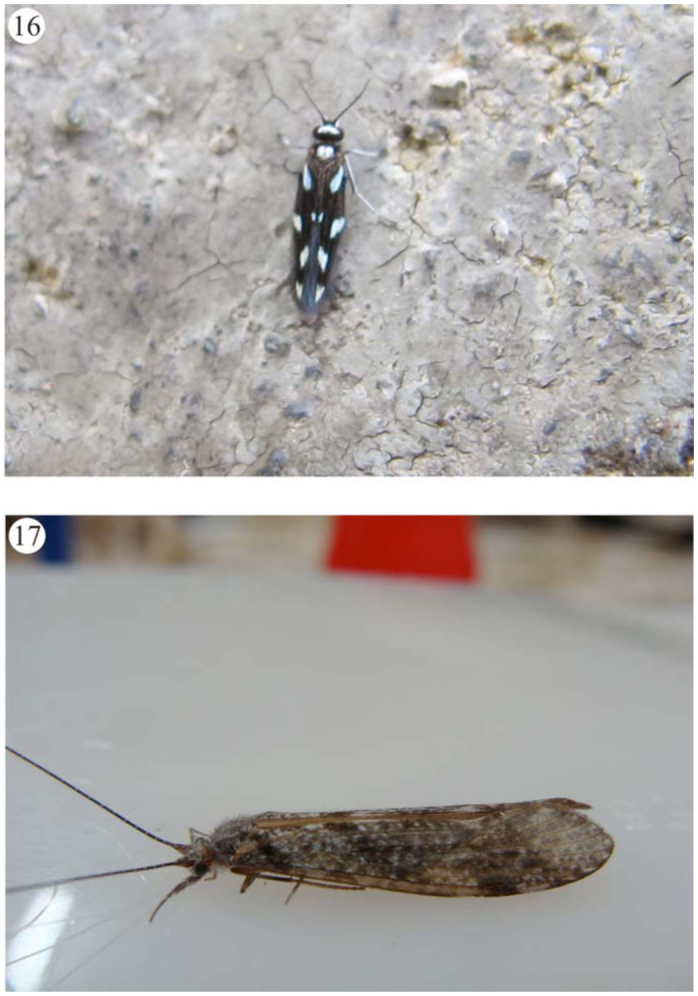
Some caddisflies specimens of Itatiaia massif. (16) *Byrsopteryx abrelata* Harris and Holzenthal (Hydroptilidae); (17) *Triplectides ultimus* Holzenthal (Leptoceridae). Picture 16 – Daniela Maeda Takiya. High quality figures are available online.

## Results

An amount of 92 species of caddisflies belonging to 35 genera were recorded from Itatiaia massif (Figures 12–17). Six additional genera were collected solely as larvae: *Alisotrichia* Flint, 1964, *Metrichia* Ross, 1938, *Ochrotrichia* Mosely, 1934 (Hydroptilidae), *Oecetis* McLachlan, 1877 (Leptoceridae), *Cyrnellus* Banks, 1913 (Polycentropodidae), and *Grumicha* Müller, 1879 (Sericostomatidae). Only six species previously recorded from Itatiaia massif were not collected in field works: *Itauara julia* Robertson and Holzenthal, 2011, *Mortoniella crescentis* Blahnik and Holzenthal, 2011, *Mortoniella latispina* Blahnik and Holzenthal, 2011 (Glossosomatidae), *Macronema partitum* Navás, 1932 (Hydropsychidae), *Austrotinodes taquaralis* Thomson and Holzenthal, 2010 (Ecnomidae), and *Polycentropus inusitatus* Hamilton and Holzenthal, 2011 (Polycentropodidae). These species were included here based on bibliography. Four species are recorded here based only on UMSP insect collection: *Atopsyche acahuana* Schmid, 1989 (Hydrobiosidae), *Smicridea iguazu* Flint, 1983 *S. radula* Flint, 1974 (Hydropsychidae), and *Nectopsyche pantosticta* Flint, 1983 (Leptoceridae). New records from the states of Minas Gerais, Rio de Janeiro, and São Paulo were previously given in Dumas et al. (2009, 2010). *Polycentropus rosalysae* Hamilton and Holzenthal, 2011 is recorded for the first time from states of Minas Gerais and Rio de Janeiro. *Polyplectropus annulicornis* Ulmer, 1905 and *P. hystricosus* Chamorro-Lacayo and Holzenthal, 2010 are recorded for the first time from state of Rio de Janeiro. Dumas et al. (2009) erroneously recorded *P. profaupar* Holzenthal and Almeida, 2003 for Itatiaia massif, state of Rio de Janeiro. Actually, the new record refers to *P. annulicornis*, as cited here.

Trichoptera families with greatest diversity in the study area are Leptoceridae (17 species), Hydropsychidae (16 species), and Philopotamidae (13 species). However, Leptoceridae and Hydropsychidae are represented by five genera each, whereas Philopotamidae is represented by only two genera. The least diverse families were Anomalopsychidae, Atriplectididae, Helicopsychidae, Limnephilidae, and Xiphocentronidae, which were represented by a single species each.

A catalog of species from Itatiaia massif with their distribution in the study area is provided below. The catalog is organized alphabetically by family, genus, and species. Each species name is followed by its author, date, and bibliographic citation of publication and page number on which the name was formally established. Information on type locality, holotype depository, and sex of the type are given in square brackets. Citations for any significant publication - redescriptions, description of immature stages, lectotype and neotype designations, checklist, and new distribution records - are also given. Synonyms are listed below the valid species name. Sampling sites where the species were collected in Itatiaia massif are given according to the codes presented in Tables 1–5. Occurrence of each species within Brazil is given in brackets on distribution section. Codes for Brazilian states are as follow: Amazonas (AM), Bahia (BA), Distrito Federal (DF), Espírito Santo (ES), Goiás (GO), Minas Gerais (MG), Pará (PA), Paraná (PR), Rio de Janeiro (RJ), Rio Grande do Sul (RS), Santa Catarina (SC), and São Paulo (SP).

### Acronyms of Holotype Depositories

**BMNH** — The Natural History Museum, London, England, United Kingdom. **CASC** — California Academy of Sciences, San Francisco, California, USA. **DEIC** — Deutsches Entomologisches Institut, Müncheberg, Germany. **DZRJ** — Coleção Entomológica Prof. José Alfredo Pinheiro Dutra, Instituto de Biologia, Universidade Federal do Rio de Janeiro, Rio de Janeiro, Brazil. **DZUP** — Coleção Entomológica Padre Jesus Santiago Moure, Departamento de Zoologia, Universidade Federal do Paraná, Paraná, Brazil. **ISNB** — Institut Royal des Sciences Naturelles de Belgique, Brussels, Belgium. **ISMA** — Instituto San Miguel, Buenos Aires, Argentina. **MACN** — Museo Argentina de Ciencias Naturales, Buenos Aires, Argentina. **MCZN** — Museum of Comparative Zoology, Harvard University, Cambridge, Massachusetts, USA. **MZBS** — Museo de Zoologia, Barcelona, Spain. **MZSP** — Museu de Zoologia, Universidade de São Paulo, São Paulo, Brazil. **NHMW** — Naturhistorisches Museum Wien, Wien, Austria. **MLUH** — Martin-Luther-Universität, Wissenschaftsbereich Zoologie, Halle an der Salle, Germany. **MZPW** — Polish Academy of Science, Museum of the Institute of Zoology, Warsaw, Poland. **UMSP** — University of Minnesota insect collection, Minnesota, USA. **USNM** — National Museum of Natural History, Washington, DC, USA. **ZMUH** — Universität von Hamburg, Zoologisches Institut und Zoologisches Museum, Hamburg, Germany. **ZSMC** — Zoologischen Staatssamlung München, Munich, Germany.

### ANOMALOPSYCHIDAE

#### 1. *Contulma tijuca* Holzenthal and Flint, 1995

*Contulma tijuca*: Holzenthal and Flint 1995: 22 [Type locality: Brazil, Rio de Janeiro, Parque Nacional da Tijuca, Represa dos Ciganos; holotype depository: MZSP; male; female; probable larva] — Paprocki, Holzenthal and Blahnik 2004: 5 [checklist] — Dumas et al. 2009: 371 [checklist].

**Sites in Itatiaia massif:** CB 07, and PE 01.

Distribution: Brazil (RJ).

### ATRIPLECTIDIDAE

#### 2. *Neoatriplectides desiderata* Dumas and Nessimian, 2008

*Neoatriplectides desiderata*: Dumas and Nessimian 2008: 64 [Type locality: Brazil, Minas Gerais, Itamonte, Rio Aiuruoca, 22°20′56.9″S 44°41′37.9″W, 1860 m; holotype depository: DZRJ; male; pupa] — Dumas et al. 2009: 371 [checklist].

*Neoatriplectides* sp.: Holzenthal 1997: 160 [larva] — Paprocki, Holzenthal and Blahnik 2004: 5 [checklist] — Dumas and Nessimian 2008: 64 [association with *N. desiderata*].

**Sites in Itatiaia massif:** AI 02, AI 04, AI 11, CB 21, and PR 12.

Distribution: Brazil (MG, RJ, SP).

### CALAMOCERATIDAE

#### 3. *Phylloicus abdominalis* (Ulmer, 1905)

*Phylloicus abdominalis*: Ulmer 1905a: 34 [Type locality: Brazil, “Are-as” [probably in Santa Catarina]; holotype depository: MLUH — type destroyed; male; in *Homoeoplectron*] — Ulmer 1905b: 77 [comb. nov., as *Phylloicus abdominalis*] — Ulmer 1913: 398 [distribution] — Prather 2003: 15 [neotype; male; female] — Paprocki, Holzenthal and Blahnik 2004: 5 [checklist] — Huamantinco, Dumas and Nessimian 2005: 20 [larva; pupa] — Dumas et al. 2009: 367 [checklist].

**Sites in Itatiaia massif:** AI 04, CB 11, CB 12, CB 13, CB 15, CB 19, CB 20, CB 21, PE 03, PE 04, PR 02, PR 03, PR 09, PR 12, and PR 14.

Distribution: Argentina, and Brazil (MG, RJ, SC, SP, PR).

#### 4. *Phylloicus angustior* Ulmer, 1905

*Phylloicus angustior:* Ulmer 1905b: 78 [Type locality: Brazil, Rio Grande do Sul; holotype depository: NHMW; male] — Flint 1966: 11 [lectotype; male] — Botosaneanu and Flint 1982: 24 [larva] — Botosaneanu and Alkins-Koo 1993: 38 [distribution] — Prather 2003: 27 [male; female] — Paprocki, Holzenthal and Blahnik 2004: 5 [checklist].

*Phylloicus hansoni*: Denning, in Denning, Resh and Hogue 1983: 184 [Type locality: Trinidad, Simla Research Station; holotype depository: CASC; male] — Botosaneanu and Alkins-Koo 1993: 38 [to synonymy].

**Sites in Itatiaia massif:** AI 04.

Distribution: Argentina, Brazil (GO, MG, PR, RS, SC), Colombia, Trinidad, and Venezuela.

#### 5. *Phylloicus bidigitatus* Prather, 2003

*Phylloicus bidigitatus*: Prather 2003: 34 [Type-locality: Brazil, Rio de Janeiro, Itatiaia; holotype depository: NHMW; male] — Paprocki, Holzenthal and Blahnik 2004: 5 [checklist] — Dumas et al. 2009 [checklist] — Dumas et al. 2010: 7 [distribution].

**Sites in Itatiaia massif:** AI 06, AI 09, CB 09, and CB 11.

Distribution: Brazil (RJ, SP).

#### 6. *Phylloicus monneorum* Dumas and Nessimian, 2010

*Phylloicus monneorum*: Dumas and Nessimian 2010a: 309 [Type locality: Brazil, Rio de Janeiro, Itatiaia, Parque Nacional do Itatiaia, Rio Campo Belo tributary, in the track to Lago Azul, 22°27′8.38″S 44°36′40.99″W, 790 m; holotype depository: DZRJ; male; female].

**Sites in Itatiaia massif:** CB 10, and CB 23.

Distribution: Brazil (RJ).

### ECNOMIDAE

#### 7. *Austrotinodes prolixus* Flint and Denning, 1989

*Austrotinodes prolixus*: Flint and Denning 1989: 120 [Type locality: Brazil, Minas Gerais, [Jaboticatubas municipality], Chapeau do Sol [sic, recte Chapéu do Sol], Km 110, Serra do Cipó; type depository: MZSP; male] — Paprocki, Holzenthal and Blahnik 2004: 5 [checklist] — Dumas et al. 2009: 357 [checklist; distribution] — Dumas et al. 2010: 7 [distribution].

**Sites in Itatiaia massif:** PE 01, PE 02, PE 03, PE 04, PE 05, PR 12, and SA 01.

Distribution: Brazil (MG, RJ, SP).

#### 8. *Austrotinodes taquaralis* Thomson and Holzenthal, 2010

*Austrotinodes taquaralis*: Thomson and Holzenthal 2010: 47 [Type locality: Brazil, Rio de Janeiro, Parque Nacional Itatiaia, Rio Taquaral, 22°27.252′S 44°36.570′W, 1300 m; holotype depository: MZSP; male].

**Sites in Itatiaia massif:** Rio de Janeiro, Itatiaia municipality, PNI, Rio Taquaral (22°24′33″S 44°33′08″W) — *see* Thomson and Holzenthal 2010.

Distribution: Brazil (MG, RJ).

### GLOSSOSOMATIDAE

#### 9. *Itauara Julia* Robertson and Holzenthal, 2011

*Itauara julia*: Robertson and Holzenthal 2011: 73 [Type locality: Brazil, Rio de Janeiro, Parque Nacional Itatiaia, Rio Campo Belo, trail to Véu da Noiva, 22°25′42″S 44°37′10″W, 1310 m; holotype depository: MZSP; male].

**Sites in Itatiaia massif:** Rio de Janeiro, Itatiaia municipality, PNI, Rio Campo Belo, trail to Véu da Noiva (22°25′42″S 44°37′10″W); PNI, Rio Campo Belo (22°27′02″S 44°36′49″W); PNI, Rio Taquaral (22°27′15″S 44°36′34″W) — *see* Robertson and Holzenthal 2011.

Distribution: Brazil (RJ).

#### 10. *Mortoniella crescentis* Blahnik and Holzenthal, 2011

*Mortoniella crescentis*: Blahnik and Holzenthal 2011: 14 [Type locality: Brazil, Rio de Janeiro, Parque Nacional Itatiaia, Rio Campo Belo, trail to Véu da Noiva, 22°25′42″S 44°37′10″W, 1310 m; holotype depository: MZSP; male].

**Sites in Itatiaia massif:** Rio de Janeiro, Itatiaia municipality, PNI, Rio Campo Belo, trail to Véu da Noiva (22°25′42″S 44°37′10″W); PNI, Rio Campo Belo (22°27′02″S 44°36′49″W) — *see* Blahnik and Holzenthal 2011.

Distribution: Brazil (RJ).

#### 11. *Mortoniella latispina* Blahnik and Holzenthal, 2011

*Mortoniella latispina*: Blahnik and Holzenthal 2011: 14 [Type locality: Brazil, Rio de Janeiro, Parque Nacional Itatiaia, Rio Campo Belo, trail to Véu da Noiva, 22°25′42″S 44°37′10″W, 1310 m; holotype depository: MZSP; male].

**Sites in Itatiaia massif:** Rio de Janeiro, Itatiaia municipality, PNI, Rio Campo Belo, trail to Véu da Noiva (22°25′42″S 44°37′10″W) — *see* Blahnik and Holzenthal 2011.

Distribution: Brazil (RJ).

#### 12. *Mortoniella teutona* (Mosely, 1939)

*Mortoniella teutona*: Mosely 1939a: 223 [Type locality: Brazil, Santa Catarina, [Seara municipality], Nova Teotônia [sic, recte Nova Teutônia]; holotype depository: BMNH; male; in *Mexitrichia*] — Flint 1963: 474 [distribution] — Flint 1966: 2 [erroneously to synonymy with *M. albolineata*] — Flint 1972: 226 [resurrected; distribution] — Angrisano 1997: 58 [distribution] — Blahnik, Paprocki and Holzenthal 2004: 4 [distribution] — Paprocki, Holzenthal and Blahnik 2004: 6 [checklist] — Blahnik and Holzenthal 2008: 69 [comb, nov., as *Mortoniella teutona*; in *leroda* group] — Dumas et al. 2009: 364 [checklist] — Blahnik and Holzenthal 2011: 31 [redescription; distribution].

**Sites in Itatiaia massif:** PE 02, PE 03, PE 04, and PE 05.

Distribution: Argentina, Brazil (MG, RJ, SC), and Uruguay.

### HELICOPSYCHIDAE

#### 13. *Helicopsyche (Feropsyche) monda* Flint, 1983

*Helicopsyche monda*: Flint 1983: 93 [Type locality: Paraguay, Depto. Alto Paraná, Salto del Monday, near Puerto Presidente Franco; holotype depository: USNM; male] — Johanson 2002: 92 [redescription; distribution] — Blahnik, Paprocki and Holzenthal 2004: 4 [distribution] — Paprocki, Holzenthal and Blahnik 2004: 6 [checklist] — Dumas et al. 2009: 371 [checklist; distribution].

**Sites in Itatiaia massif:** CB 13, PR 12, and PR 14.

Distribution: Argentina, Brazil (MG, PR, RJ, SC, SP), and Paraguay.

### HYDROBIOSIDAE

#### 14. *Atopsyche (Atopsaura) hatunpuna* Schmid, 1989

*Atopsyche hatunpuna*: Schmid 1989: 122 [Type locality: Brazil, São Paulo, [Salesópolis municipality], Casa Grande, Ribeirão Coruja; holotype depository: MZSP; male; in *longipennis* group] — Paprocki, Holzenthal and Blahnik 2004: 7 [checklist] — Dumas et al. 2010: 7 [distribution].

**Sites in Itatiaia massif:** AI 03, and AI 10. Distribution: Brazil (MG, SP).

#### 15. *Atopsyche (Atopsaura) huamachucu* Schmid, 1989

*Atopsyche huamachucu*: Schmid 1989: 124 [Type locality: Brazil, Rio de Janeiro, Km 17, 18 Km S of Teresópolis; holotype depository: MZSP; male; in *longipennis* group] — Paprocki, Holzenthal and Blahnik 2004: 7 [checklist] — Dumas et al. 2009: 365 [checklist].

**Sites in Itatiaia massif:** CB 02, and CB 05.

Distribution: Brazil (RJ).

#### 16. *Atopsyche (Atopsaura) huanapu* Schmid, 1989

*Atopsyche huanapu*: Schmid 1989: 124 [Type locality: Brazil, São Paulo, [Salesópolis municipality], E.B. de Boracéia, Parede da Pedreira; holotype depository: MZSP; male; in *longipennis* group] — Blahnik, Paprocki and Holzenthal 2004: 4 [distribution] — Paprocki, Holzenthal and Blahnik 2004: 7 [checklist] — Dumas et al. 2009: 365 [checklist].

**Sites in Itatiaia massif:** CB 02, CB 21, PE 04, and PR 12.

Distribution: Brazil (RJ, SP).

#### 17. *Atopsyche (Atopsaura) huarcu* Schmid, 1989

*Atopsyche huarcu*: Schmid 1989: 125 [Type locality: Brazil, Minas Gerais, Nova Lima; holotype depository: MZSP; male; in *longipennis* group] — Blahnik, Paprocki and Holzenthal 2004: 4 [distribution] — Paprocki, Holzenthal and Blahnik 2004: 7 [checklist] — Dumas et al. 2009: 365 [checklist].

**Sites in Itatiaia massif:** CB 02, CB 13, and PE 04.

Distribution: Brazil (MG, RJ, SP).

#### 18. *Atopsyche (Atopsaura) sanctipauli* Flint, 1974

*Atopsyche sanctipauli*: Flint 1974a: 5 [Type locality: Brazil, São Paulo, Alto da Serra [probably Santo André municipality, Paranapiacaba village]; holotype depository: NHMW; male] — Schmid 1989: 144 [in *longipennis* group] — Blahnik, Paprocki and Holzenthal 2004: 4 [distribution] — Paprocki, Holzenthal and Blahnik 2004: 7 [checklist] — Dumas et al. 2009: 365 [checklist].

**Sites in Itaiaia massif:** PE 01, and PE 05.

Distribution: Brazil (RJ, PR, SC, SP).

#### 19. *Atopsyche (Atopsaura) zernyi* Flint, 1974

*Atopsyche zernyi*: Flint 1974a: 5 [Type locality : Brazil, São Paulo, Alto da Serra [probably Santo André municipality, Paranapiacaba village]; holotype depository: NHMW; male] — Schmid 1989: 144 [in *longipennis* group] — Blahnik, Paprocki and Holzenthal 2004: 4 [distribution] — Paprocki, Holzenthal and Blahnik 2004: 7 [checklist] — Dumas et al. 2009: 365 [checklist] — Dumas et al. 2010: 7 [distribution].

**Sites in Itatiaia massif:** AI 03, CB 07, CB 14, PR 09, PR 10, and SA 04.

Distribution: Brazil (ES, MG, RJ, SC, SP).

### HYDROPSYCHIDAE

#### 20. *Centromacronema auripenne* (Rambur, 1842)

*Centromacronema auripenne*: Rambur 1842: 507 [Type locality: Brazil; holotype depository: ISNB; male ; in *Macronema*] — Ulmer 1905b: 87 [comb. nov., as *Centromacronema auripenne*] — Ulmer 1907a: 63 [type species] — Ulmer 1907b: 112 [male; redescription] — Betten and Mosely 1940: 209 [type redescription of *C. cupreum*; venation] — Holzenthal 1988a: 65 [distribution] — Flint 1996: 411 [distribution] — Paprocki, Holzenthal and Blahnik 2004: 7 [checklist] — Dumas et al. 2009: 357 [checklist].

*Macronema cupreum*: Walker 1852: 76 [Type locality: Brazil; holotype depository: BMNH; male] — Ulmer 1907b: 112 [to synonymy].

*Leptocerus niveistigma*: Walker 1860: 176 [Type locality: Brazil; holotype depository: BMNH; male] — Ulmer 1907b: 112 [to synonymy].

*Leptocerus abjurans*: Walker 1860: 177 [Type locality: Brazil; holotype depository: BMNH; male] — Ulmer 1907b: 112 [to synonymy].

*Leptocerus quadrifurcata*: Walker 1860: 177 [Type locality: Brazil; holotype depository: BMNH; male] — Ulmer 1907b: 112 [to synonymy].

*Centromacronema extensum* Banks 1913: 238 [Type locality: Panama, Lino; holotype depository: MCZN; male] — Flint 1967: 7 [to synonymy].

**Sites in Itatiaia massif:** AI 04, PE 01, PE 03, and PR 06.

Distribution: Bolivia, Brazil (MG, RJ, SC, SP), Colombia, Costa Rica, El Salvador, Guatemala, Guyana, Honduras, Mexico, Nicaragua, Panama, Peru, and Venezuela.

#### 21. *Leptonema bifurcatodes* Flint, 2008

*Leptonema bifurcatodes*: Flint 2008: 462 [Type locality: Brazil, Rio de Janeiro, Parque Nacional do Itatiaia, Rio Campo Belo, 22°27.033′N 44°36.318′W; holotype depository: MZSP, male] — Dumas et al. 2009: 357 [checklist].

**Sites in Itatiaia massif:** CB 11.

Distribution: Brazil (RJ).

#### 22. *Leptonema pallidum* Guérin, 1843

*Leptonema pallidum*: Guérin 1843: 396 [Type locality: Brazil; holotype depository: unknown; sex undetermined] — Flint, McAlpine and Ross 1987: 68 [male; distribution] — Oliveira and Froehlich 1996: 757 [biology] — Paprocki, Holzenthal and Blahnik 2004: 7 [checklist] — Dumas et al. 2009: 358 [checklist].

*Leptonema furcatum*: Ulmer 1905a: 57 [Type locality: Brazil, Espírito Santo; holotype depository: MZPW; male] — Mosely 1939b: 310 [to synonymy].

*Hydropsyche flagellata*: Jacquemart 1962: 6 [Type locality: Brazil, Rio de Janeiro, Bomanca; holotype depository: ISNB; male] — Flint, McAlpine and Ross 1987: 68 [to synonymy].

**Sites in Itatiaia massif:** PE 02, and SA 03.

Distribution: Argentina and Brazil (DF, ES, GO, MG, RJ, SP).

#### 23. *Leptonema tridens* Mosely, 1933

*Leptonema tridens*: Mosely 1933: 17 [Type locality: Brazil, Paraná; holotype depository: BMNH; male] — Flint, McAlpine and Ross 1987: 46 [male; distribution] — Blahnik, Paprocki and Holzenthal 2004: 4 [distribution] — Paprocki, Holzenthal and Blahnik 2004: 8 [checklist] — Dumas et al. 2009: 357 [checklist] — Nessimian and Dumas 2010: 466 [larva; pupa].

**Sites in Itatiaia massif:** AI 01, AI 04, AI 10, PR 02, PR 04, PR 08, PR 10, PR 12, and PR 14.

Distribution: Brazil (MG, PR, RJ, SP), and Paraguay [?].

#### 24. *Leptonema viridianum* Navás, 1916

*Leptonema viridianum*: Navás 1916a: 31 [Type locality: Brazil, Bahia; holotype depository: collection Navás, now lost; female] — Flint, McAlpine and Ross 1987: 70 [male; distribution] — Oliveira and Froehlich 1996: 757 [biology] — Blahnik, Paprocki and Holzenthal 2004: 4 [distribution] — Paprocki, Holzenthal and Blahnik 2004: 7 [checklist] — Dumas et al. 2009: 358 [checklist] — Dumas et al. 2010: 8 [distribution].

*Leptonema dissimile*: Mosely 1933: 43 [Type locality: Bolivia, Pcia. Sara; holotype depository: MCZN; male] — Flint, 1978: 384 [to synonymy].

**Sites in Itatiaia massif:** PE 05.

Distribution: Argentina, Bolivia, Brazil (BA, MG, RJ, PR, PA, SP), Colombia, Ecuador, Guyana, Paraguay, Peru, and Venezuela.

#### 25. *Macronema bicolor* Ulmer, 1905

*Macronema bicolor*: Ulmer 1905a: 75 [Type locality: Brazil, Santa Catarina; holotype depository: MZPW; male] — Flint 1966: 6 [male; lectotype; wings] — Flint and Bueno-Soria 1982: 359 [male; synonymy; wings] — Paprocki, Holzenthal and Blahnik 2004: 8 [checklist] — Dumas et al. 2010: 8 [distribution].

*Macronema agnathum*: Müller 1921: 530 [Type locality: unknown, but presumably Brazil, Santa Catarina; holotype depository: NHMW; male] — Flint and Bueno-Soria 1982: 359 [to synonymy].

*Leptonema apicale*: Navás 1927: 40 [Type locality: Brazil, Minas Gerais; holotype depository: DEIC; male] — Flint and Bueno-Soria 1982: 359 [to synonymy].

**Sites in Itatiaia massif:** AI 08, and AI 10.

Distribution: Brazil (MG, SC, SP).

#### 26. *Macronema partitum* Navás, 1932

*Macronema partitum*: Navás 1932: 63 [Type locality: Brazil, Rio de Janeiro, Barão Homem de Melo [currently Itatiaia municipality]; holotype depository: DEIC; female] — Paprocki, Holzenthal and Blahnik 2004: 8 [checklist] — Dumas et al. 2009: 358 [checklist].

**Sites in Itatiaia massif:** Rio de Janeiro, Barão Homem de Mello [currently Itatiaia municipality] — *see* Navás 1932.

Distribution: Brazil (RJ).

#### 27. *Macrostemum hyalinum* (Pictet, 1836)

*Macrostemum hyalinum*: Pictet 1836: 401 [Type locality: Indes Orientalis; holotype depository: unknown; sex undetermined; as *Hydropsyche hyalina*] — Hagen 1861: 328 [comb, nov., as *Macronema hyalina*] — Ulmer 1907b: 75 [wings] — Flint 1978: 389 [male; wings] — Flint and Bueno-Soria 1982: 358 [comb, nov., as *Macrostemum hyalinum*] — Flint 1996: 412 [distribution] — Marinoni and Almeida 2000: 379 [distribution] — Paprocki, Holzenthal and Blahnik 2004: 8 [distribution] — Dumas et al. 2009: 358 [checklist; distribution].

**Sites in Itatiaia massif:** CB 16, and SA 03.

Distribution: Brazil (PA, PR, RJ), Colombia, Guyana, Peru, and Venezuela.

#### 28. *Macrostemum maculatum* (Perty, 1833)

*Macrostemum maculatum*: Perty 1833: 129 [Type locality: Brazil, [São Paulo], inter St. Pauli civitatem et Villain Riccam; holotype depository: ZSMC; male; as *Phryganea maculata*] — Ulmer 1905b: 82 [comb, nov., as *Macronema maculata*] — Burmeister 1983: 273 [lectotype] — Burmeister 1989: 259 [description of lectotype] — Flint and Bueno-Soria 1982: 358 [comb. nov., as *Macrostemum maculatum*] — Paprocki, Holzenthal and Blahnik 2004: 8 [checklist].

*Macronema tuberosum*: Ulmer 1905b: 82 [Type locality: Brazil, Bahia; holotype depository: NHMW; male] — Flint 1966: 7 [male; lectotype; wings] — Burmeister 1983: 273 [to synonymy].

**Sites in Itatiaia massif:** AI 08.

Distribution: Brazil (BA, MG, SP).

#### 29. *Smicridea (Smicridea) albosignata* Ulmer, 1907

*Smicridea albosignata*: Ulmer 1907c: 34 [Type locality: Brazil, Santos; holotype depository: ZMUH; male] — Weidner 1964: 97 [lectotype] — Denning and Sykora 1968: 176 [male; redescription] — Marinoni and Almeida 2000: 286[distribution] — Blahnik, Paprocki and Holzenthal 2004: 4 [distribution] — Paprocki, Holzenthal and Blahnik 2004: 10 [checklist] — Dumas et al. 2009: 359 [checklist].

**Sites in Itatiaia massif:** AI 08, CB 11, CB 09, CB 12, CB 15, PE 01, PE 04, PE 05, PR 14, SA 03, and SA 04.

Distribution: Brazil (MG, PR, RJ, SP).

#### 30. *Smicridea (Rhyacophylax) froehlichi* Almeida and Flint, 2002

*Smicridea froehlichi*: Almeida and Flint 2002: 768 [Brazil, Rio de Janeiro, Km 17, 18 Km S of Teresópolis, 1180 m; holotype depository: MZSP; male; female] — Paprocki, Holzenthal and Blahnik 2004: 9 [checklist] — Dumas et al. 2009: 359 [checklist] — Dumas et al. 2010: 8 [distribution].

**Sites in Itatiaia massif:** AI 04, CB 06, CB 07, CB 12, CB 15, CB 21, PR 04, PR 06, PR 07, PR 08, PR 09, PR 10, PR 12, PR 14, PE 01, PE 02, PE 03, PE 04, SA 01, SA 02, SA 03, SA 04, and SA 05.

Distribution: Brazil (MG, RJ, SP).

#### 31. *Smicridea (Smicridea) gemina* Blahnik, 1995

*Smicridea gemina*: Blahnik 1995: 90 [Type locality: Costa Rica, Alahuela, Reserva Florestal San Ramón, Río San Lorencito and tribs., 10.216°N 84.607°W; holotype depository: USNM; male] — Dumas et al. 2009: 359 [checklist; distribution].

**Sites in Itatiaia massif:** AI 04, PR 12, PR 14, SA 03, and SA 04.

Distribution: Brazil (MG, RJ), Costa Rica, Ecuador, Nicaragua, and Panama.

#### 32. *Smicridea (Rhyacophylax) jundiai* Almeida and Flint, 2002

*Smicridea jundiai*: Almeida and Flint 2002: 769 [Type locality: Brazil, Espírito Santo, 15 km SE of Santa Teresa, Fazenda Santa Clara, 460 m; holotype depository: MZSP; male; female] — Blahnik, Paprocki and Holzenthal 2004: 4 [distribution] — Paprocki, Holzenthal and Blahnik 2004: 9 [checklist] — Dumas et al. 2009: 360 [checklist] — Dumas et al. 2010: 8 [distribution].

**Sites in Itatiaia massif:** PE 02, PE 03, PE 04, PE 05, PR 12, PR 14, and SA 01.

Distribution: Brazil (ES, MG, PR, RJ, SP).

### HYDROPTILIDAE

#### 33. *Abtrichia squamosa* Mosely, 1939

*Abtrichia squamosa*: Mosely 1939a: 226 [Type locality:Brazil, Santa Catarina, [Seara municipality], Nova Teotôônia [sic, recte Nova Teutônia]; holotype depository: BMNH; male] — Blahnik, Paprocki and Holzenthal 2004: 4 [distribution] — Paprocki, Holzenthal and Blahnik 2004: 10 [checklist] — Dumas et al. 2009: 365 [checklist].

**Sites in Itatiaia massif:** PE 02, and PE 03.

Distribution: Brazil (MG, RJ, SC).

#### 34. *Byrsopteryx abrelata* Harris and Holzenthal, 1994

*Byrsopteryx abrelata*: Harris and Holzenthal 1994: 157 [Type locality: Brazil, Rio de Janeiro, Nova Friburgo, municipal water supply, 950 m; holotype depository: MZSP; male; female] — Paprocki, Holzenthal and Blahnik 2004: 11 [checklist] — Dumas et al. 2009: 366 [checklist] — Santos and Nessimian 2010: 52 [larva; pupa; distribution].

**Sites in Itatiaia massif:** SA 04.

Distribution: Brazil (PR, RJ).

#### 35. *Hydroptila argentinica* Flint, 1983

*Hydroptila argentinica*: Flint 1983: 43 [Type locality: Argentina, Pcia. Tucumán, S. Concepición; holotype depository: USNM; male; female] — Blahnik, Paprocki and Holzenthal 2004: 5 [distribution] — Paprocki, Holzenthal and Blahnik 2004: 11 [checklist] — Dumas et al. 2009: 366 [checklist].

**Sites in Itatiaia massif:** PE 02, PE 03, PE 04, PE 05, PR 01, PR 05, PR 06, PR 07, PR 08, PR 09, PR 10, SA 01, SA 02, and SA 03.

Distribution: Argentina, Brazil (PR, RJ, SP), and Uruguay.

#### 36. *Hydroptila producta* Mosely, 1939

*Hydroptlia producta*: Mosely 1939a: 236 [Type locality: Brazil, Santa Catarina, [Seara municipality], Nova Teotônia [sic, recte Nova Teutônia]; holotype depository: BMNH; male] — Angrisano 1995b: 509 [distribution] — Paprocki, Holzenthal and Blahnik 2004: 11 [checklist] — Dumas et al. 2009: 366 [checklist; distribution].

**Sites in Itatiaia massif:** PE 02, and PE 03.

Distribution: Brazil (RJ, SC), and Uruguay.

#### 37. *Neotrichia dubitans* (Mosely, 1939)

*Neotrichia dubitans*: Mosely 1939a: 235 [Type locality: Brazil, Santa Catarina, [Seara municipality], Nova Teotônia [sic, recte Nova Teutônia]; holotype depository: BMNH; male; in *Dolotrichia*?] — Ross 1944: 154 [comb. nov., as *Neotrichia dubitans*] — Paprocki, Holzenthal and Blahnik 2004: 11 [checklist] — Dumas et al. 2009: 366 [checklist; distribution].

**Sites in Itatiaia massif:** CB 07, PE 01, PE 03, and SA 03.

Distribution: Brazil (RJ, SC).

#### 38. *Oxyethira (Loxotrichia) tica* Holzenthal and Harris, 1992

*Oxyethira tica*: Holzenthal and Harris 1992: 168 [Type locality: Costa Rica, Guanacaste, Parque Nacional Santa Rosa, Quebrada El Duende near La Casona, 10.838°N 85.614°W; holotype depository: USNM; male; female] — Flint 1996: 98 [distribution] — Blahnik, Paprocki and Holzenthal 2004: 5 [distribution] — Paprocki, Holzenthal and Blahnik 2004: 12 [checklist] — Santos, Henriques-Oliveira and Nessimian 2009: 36 [distribution] — Dumas et al. 2009: 366 [checklist; distribution].

**Sites in Itatiaia massif:** PE 03.

Distribution: Brazil (AM, MG, RJ), Costa Rica, Dominica, Ecuador, Grenada, Guadeloupe, Honduras, Mexico, Panama, St. Lucia, St. Vicent, Trinidad, and Venezuela.

#### 39. *Rhyacopsyche bulbosa* Wasmund and Holzenthal, 2007

*Rhyacopsyche bulbosa*: Wasmund and Holzenthal 2007: 8 [Type locality: Brazil, Rio de Janeiro, Nova Friburgo, municipal water supply, 950 m; holotype depository: MZSP; male] — Dumas et al. 2009: 366 [checklist; distribution].

**Sites in Itatiaia massif:** SA 04.

Distribution: Brazil (MG, RJ, SP).

#### 40. *Rhyacopsyche dikrosa* Wasmund and Holzenthal, 2007

*Rhyacopsyche dikrosa*: Wasmund and Holzenthal 2007: 11 [Type locality: Brazil, São Paulo, Pedregulho, 140 km NE Ribeirão Preto; holotype depository: MZSP; male] — Dumas et al. 2009: 366 [checklist; distribution].

**Sites in Itatiaia massif:** PE 01, PE 02, PE 03, PE 04, and SA 03.

Distribution: Brazil (MG, SP).

#### 41. *Rhyacopsyche hagenii* Müller, 1879

*Rhyacopsyche hagenii*: Müller 1879: 143 [Type locality: Brazil; holotype depository: unknown; case] — Thienemann 1905a: 287 [male; larva] — Müller 1921:525 [larva] — Ulmer 1957: 172, 187 [bibliography; key to larvae] — Angrisano 1995b: 509 [distribution] — Paprocki, Holzenthal and Blahnik 2004: 12 [checklist] — Wasmund and Holzenthal 2007: 6 [male; female; distribution] — Dumas et al. 2009: 367 [checklist].

**Sites in Itatiaia massif:** CB 04, CB 06, CB 07, CB 12, PE 01, PE 02, PE 04, and PE 05.

Distribution: Argentina, Brazil (PR, RJ, SC, SP), and Uruguay.

### LEPTOCERIDAE

#### 42. *Grumichella rostrata* Thienemann, 1905

*Grumichella rostrata*: Thienemann 1905b: 537 [Type locality: not designated [probably Brazil, Santa Catarina, Gruta dos Macacos, near Blumenau — *see* Holzenthal 1988b]; holotype depository: unknown; pupa; case] — Thienemann 1909: 41, 42, 125 [larva; pupa] — Holzenthal 1988b: 93 [male; female; larva; pupa] — Paprocki, Holzenthal and Blahnik 2004: 12 [checklist] — Dumas et al. 2009: 368 [checklist].

**Sites in Itatiaia massif:** AI 04, CB 12, PE 02, PE 03, PE 05, PR 12, and PR 14.

Distribution: Brazil (MG, RJ, SP, SC).

#### 43. *Nectopsyche aureovittata* Flint, 1983

*Nectopsyche aureovittata*: Flint 1983: 74 [Type locality: Argentina, Pcia. Misiones, Rio Iguazú, Camp Naãdu; holotype depository: USNM; male] — Almeida and Marinoni 2000: 349 [distribution] — Blahnik, Paprocki and Holzenthal 2004: 5 [distribution] — Paprocki, Holzenthal and Blahnik 2004: 12 [checklist] — Dumas et al. 2009: 368 [checklist].

**Sites in Itatiaia massif:** PR 12, PR 14, SA 01, SA 03, and SA 04.

Distribution: Argentina, Brazil (MG, PR, RJ, SC, SP), and Paraguay.

#### 44. *Nectopsyche bruchi* (Navás, 1920)

*Nectopsyche bruchi*: Navás 1920: 66 [Type locality: Argentina, Monte Veloz, estancia Barreto; holotype depository: MACN; male; in *Leptocella*] — Flint 1972: 243 [diagnosis; distribution] — Flint 1974b: 127 [comb. nov., as *Nectopsyche bruchi*] — Flint 1982: 55 [redescription; distribution] — Paprocki, Holzenthal and Blahnik 2004: 12 [checklist] — Dumas et al. 2009: 368 [checklist; distribution].

**Sites in Itatiaia massif:** PE 01.

Distribution: Argentina, Brazil (MG, PR, RJ), and Paraguay.

#### 45. *Nectopsyche fuscomaculata* Flint, 1983

*Nectopsyche fuscomaculata*: Flint 1983: 73 [Type locality: Argentina, Pcia. Misiones, Arroyo Liso, 8 km W General Güemes; holotype depository: USNM; male] — Almeida and Marinoni 2000: 349 [distribution] — Blahnik, Paprocki and Holzenthal 2004: 5 [distribution] — Paprocki, Holzenthal and Blahnik 2004: 12 [checklist] — Dumas et al. 2009: 368 [checklist; distribution].

**Sites in Itatiaia massif:** CB 12, PE 01, and PE 05.

Distribution: Argentina, Brazil (PR, RJ, SC), and Paraguay.

#### 46. *Nectopsyche muhni* (Navás, 1916)

*Nectopsyche muhni*: Navás 1916b: 68 [Type locality: Argentina, Santa Fé; holotype depository: MZBS; female; in *Leptocella*] — Schmid 1949: 388 [male] — Flint 1974b: 127 [comb. nov., as *Nectopsyche muhni*] — Flint 1982: 58 [distribution] — Blahnik, Paprocki and Holzenthal 2004: 5 [distribution] — Paprocki, Holzenthal and Blahnik 2004: 13 [checklist] — Dumas et al. 2009: 368 [checklist; distribution].

*Leptocella fulvocapilla*: Navás 1922: 399 [Type locality: Argentina, La Plata, Palo Blanco; holotype depository: MACN; male] — Flint 1972: 243 [to synonymy].

*etodes pretiosella*: Banks 1924: 447 [Type locality: Peru, Yurimaguas; holotype depository: MCZN; female] — Flint 1982: 58 [to synonymy].

*Leptocella bridarollia*: Navás 1930: 75 [Type locality: Argentina, Santa Fé; holotype depository: ISMA; female] — Flint 1982: 58 [to synonymy].

**Sites in Itatiaia massif:** CB 06, PE 02, and PR 12.

Distribution: Argentina, Bolivia, Brazil (MG, RJ), Ecuador, Guyana, Paraguay, Peru, Surinam, and Venezuela.

#### 47. *Nectopsyche ortizi* Holzenthal, 1995

*Nectopsyche ortizi*: Holzenthal 1995: 73 [Type locality: Costa Rica, Limón, Parque Nacional Tortuguero, Río Tortuguero, 3.5 Km S Tortuguero, 10.509°N 83.504°W; holotype depository: USNM; male; in *gemma* group] — Flint 1974b: 129 [male; as *N gemma*, nec Müller 1880] — Almeida and Marinoni 2000: 349 [distribution] — Blahnik, Paprocki and Holzenthal 2004: 5 [distribution] — Paprocki, Holzenthal and Blahnik 2004: 13 [checklist] — Dumas et al. 2009: 369 [checklist].

**Sites in Itatiaia massif:** AI 04, CB 07, CB 12, PE 02, PE 04, PE 05, and PR 09.

Distribution: Argentina, Brazil (MG, PA, PR, RJ, SP), Costa Rica, Guyana, Mexico, Panama, Paraguay, Peru, Surinam, and Venezuela.

#### 48. *Nectopsyche punctata* (Ulmer, 1905)

*Nectopsyche punctata*: Ulmer 1905b: 75 [Type locality: Brazil, [Minas Gerais], Santa Rita [Santa Rita de Jacutinga municipality], Boquero, Rio Preto; holotype depository: NHMW; male; in *Leptocella*] — Flint 1966: 9 [male; lectotype] — Flint 1974b: 127 [comb. nov., as *Nectopsyche punctata*] — Flint 1991: 94 [distribution] — Blahnik, Paprocki and Holzenthal 2004: 5 [distribution] — Paprocki, Holzenthal and Blahnik 2004: 13 [checklist] — Dumas et al. 2009: 369 [checklist; distribution].

*Leptocella fenestrata*: Banks 1913: 237 [Type locality: Panama, Lino; holotype depository: MCZN; male] — Flint 1966: 9 [to synonymy].

*Leptocella spegazzinia*:Navás 1920: 69 [Type locality: Paraguay, Rio Paraguay; holotype depository: MZBS; male] — Flint 1981: 34 [to synonymy].

*Leptocella ambitiosa*: Navás 1933: 118 [Type locality: Argentina, Santa Fé; holotype depository: MZBS; male] — Schmid 1949: 386 [to synonymy with *N. mixta*] — Flint 1966: 9 [to synonymy].

**Sites in Itatiaia massif:** CB 11, PE 03, SA 02, and SA 03.

Distribution: Argentina, Bolivia, Brazil (MG, PA, RJ, SP), Colombia, Costa Rica, Ecuador, Guyana, Mexico, Panama, Paraguay, Peru, Surinam, and Venezuela.

#### 49. *Nectopsyche separata* (Banks, 1920)

*Nectopsyche separata*: Banks 1920: 353 [Type locality: Brazil, Santa Catarina; holotype depository: MCZN; male; in *Leptocella*] — Flint 1967: 22 [male; lectotype] — Flint 1972: 242 [distribution] — Flint 1974b: 127 [comb. nov., as *Nectopsyche separata*] — Almeida and Marinoni 2000: 349 [distribution] — Blahnik, Paprocki and Holzenthal 2004: 5 [distribution] — Paprocki, Holzenthal and Blahnik 2004: 13 [checklist] — Dumas et al. 2009: 369 [checklist].

*Leptocella graphica*: Navás 1932: 65 [Type locality: Brazil, Rio de Janeiro, Barão Homem de Melo [currently Itatiaia municipality]; holotype depository: DEIC; male] — Flint 1982: 59 [to synonymy].

**Sites in Itatiaia massif:** CB 15, PE 04, PR 04, PR 07, PR 08, PR 09, PR 10, and SA 02.

Distribution: Argentina, Brazil (MG, PR, RJ, SC, SP), and Paraguay.

#### 50. *Neoathripsodes anomalus* Holzenthal, 1989

*Neoathripsodes anomalus*: Holzenthal 1989: 31 [Type locality: Brazil, Rio de Janeiro, Km 17, 18 Km S Teresópolis; holotype depository: MZSP; male] — Blahnik, Paprocki and Holzenthal 2004: 5 [distribution] — Paprocki, Holzenthal and Blahnik 2004: 13 [checklist] — Dumas et al. 2009: 369 [checklist].

**Sites in Itatiaia massif:** AI 06, AI 08, CB 13, CB 20, PE 05, and SA 03.

Distribution: Brazil (MG, RJ).

#### 51. *Notalina (Neonotalina) hamiltoni* Holzenthal, 1986

*Notalina hamiltoni*: Holzenthal 1986: 67 [Type locality: Brazil, São Paulo, [Salesópolis municipality], E.B. Boracéia; holotype depository: MZSP; male] — Paprocki, Holzenthal and Blahnik 2004: 13 [checklist] — Dumas et al. 2009: 370 [checklist; distribution] — Dumas et al. 2010: 8 [distribution].

**Sites in Itatiaia massif:** AI 04, and PE 04.

Distribution: Brazil (MG, RJ, SP).

#### 52. *Notalina (Neonotalina) morsei* Holzenthal, 1986

*Notalina morsei*: Holzenthal 1986: 63 [Type locality: Brazil, Minas Gerais, Serra do Cipó; holotype depository: MZSP; male] — Calor, Holzenthal and Amorim 2007: 42 [phylogeny; distribution] — Paprocki, Holzenthal and Blahnik 2004: 13 [checklist] — Calor and Froehlich 2008: 46 [larva; pupa] — Dumas et al. 2009: 370 [checklist].

**Sites in Itatiaia massif:** CB 06, CB 07, CB 11, CB 12, CB 13, CB 15, PE 01, PE 04, PE 05, PR 09, PR 12, SA 03, and SA 04.

Distribution: Brazil (MG, RJ, SP).

#### 53. *Triplectides gracilis* (Burmeister, 1839)

*Triplectides gracilis*: Burmeister 1839: 921 [Type locality: Brazil, Nova Friburgo; holotype depository: MLUH — type destroyed; male; in *Mystacides*] — Ulmer 1905a: 27 [redescription of male type; comb. nov., as *Triplectides gracilis*] — Mosely 1936: 96 [male] — Holzenthal 1988c: 195 [Neotype: Brazil, Rio de Janeiro, Nova Friburgo, municipal water supply; neotype depository: USNM; male; female; redescription; distribution] — Almeida and Marinoni 2000: 349 [distribution] — Paprocki, Holzenthal and Blahnik 2004: 13 [checklist] — Dumas et al. 2009: 370 [checklist].

*Mystacides princeps*: Burmeister 1839: 921 [Type locality: Brazil, Rio de Janeiro, Nova Friburgo; holotype depository: MLUH — type destroyed; male] — Ulmer 1905a: 27 [to synonymy].

*Tetracentron ramulorus*: Müller 1921: 541 [Type locality: Brazil, Santa Catarina; holotype depository: unknown; larva; pupa] — Holzenthal 1988c: 195 [to synonymy].

**Sites in Itatiaia massif:** AI 04, AI 06, CB 07, CB 08, CB 11, CB 12, CB 13, CB 16, CB 21, PE 01, PE 03, PE 04, PR 03, PR 05, PR 09, PR 10, PR 12, PR 14, SA 03, and SA 05.

Distribution: Argentina, Brazil (ES, MG, PR, RJ, SC, SP), Paraguay, and Surinam.

#### 54. *Triplectides itatiaia* Dumas and Nessimian, 2010

*Triplectides itatiaia*: Dumas and Nessimian 2010b: 949 [Type locality: Brazil, Rio de Janeiro, Itatiaia, Parque Nacional do Itatiaia, Rio Tapera, 22°26′59.64″ S 44°36′19.39″ W, 794 m; holotype depository: DZRJ; male].

**Sites in Itatiaia massif:** CB 08, and CB 20.

Distribution: Brazil (RJ).

#### 55. *Triplectides misionensis* Holzenthal, 1988

*Triplectides misionensis*: Holzenthal 1988c: 198 [Type locality: Argentina, Misiones, Arroyo Piray Guazú, N San Pedro; holotype depository: USNM; male] — Blahnik, Paprocki and Holzenthal: 5 [distribution] — Paprocki, Holzenthal and Blahnik 2004: 13 [checklist] — Dumas et al. 2009: 371 [checklist] — Dumas et al. 2010: 8 [distribution].

**Sites in Itatiaia massif:** AI 06, and SA 05.

Distribution: Argentina, and Brazil (MG, PR, RJ, SC, SP).

#### 56. *Triplectides neotropicus* Holzenthal, 1988

*Triplectides neotropicus*: Holzenthal 1988c: 200 [Type locality: Venezuela, Territorio Federal Amazonas, camp IV, Cerro de la Neblina, 0°58′N 65°57′W; holotype depository: USNM; male] — Blahnik, Paprocki and Holzenthal: 5 [distribution] — Paprocki, Holzenthal and Blahnik 2004: 13 [checklist] — Dumas et al. 2009: 371 [checklist; distribution] — Dumas et al. 2010: 8 [distribution].

**Sites in Itatiaia massif:** CB 07.

Distribution: Brazil (MG, RJ, SP), and Venezuela.

#### 57. *Triplectides ultimus* Holzenthal, 1988

*Triplectides ultimus*: Holzenthal 1988c: 205 [Type locality: Brazil, Rio de Janeiro, Itatiaia; holotype depository: MZSP; male; female] — Blahnik, Paprocki and Holzenthal: 5 [distribution] — Paprocki, Holzenthal and Blahnik 2004: 13 [checklist] — Dumas et al. 2009: 371 [checklist] — Dumas et al. 2010: 8 [distribution].

**Sites in Itatiaia massif:** AI 04, AI 08, AI 09, AI 10, CB 15, PR 03, PR 14, and SA 03.

Distribution: Brazil (MG, RJ).

### LIMNEPHILIDAE

#### 58. *Antarctoecia brasiliensis* Huamantinco and Nessimian, 2003

*Antarctoecia brasiliensis*: Huamantinco and Nessimian 2003: 226 [Type locality: Brazil, Minas Gerais, Itamonte, Rio Aiuruoca, 22°20′9.28″S 44°41′6.06″W, 1860 m; holotype depository: DZRJ; male; female] — Huamantinco and Nessimian 2004a: 2 [larva; pupa] — Paprocki, Holzenthal and Blahnik 2004: 13 [checklist].

**Sites in Itatiaia massif:** AI 01, AI 02, and AI 04.

Distribution: Brazil (MG).

### ODONTOCERIDAE

#### 59. *Anastomoneura guahybae* Huamantinco and Nessimian, 2004

*Anastomoneura guahybae*: Huamantinco and Nessimian 2004b: 282 [Type locality: Brazil, Minas Gerais, Itamonte, Rio Aiuruoca, 22°20′9.28″S 44°41′6.06″W, 1860 m; holotype depository: DZRJ; male; female] — Dumas and Nessimian 2006: 45 [larva; pupa; distribution].

**Sites in Itatiaia massif:** AI 04, AI 08, AI 09, and AI 11.

Distribution: Brazil (MG).

#### 60. *Barypenthus concolor* Burmeister, 1839

*Barypenthus concolor*: Burmeister 1839: 929 [Type locality: Brazil, Rio de Janeiro, Nova Friburgo; holotype depository: MLUH — type destroyed; male] — Ulmer 1905a: 22 [male] — Paprocki and Holzenthal 2002: 224 [redescription; male; wings; biology] — Paprocki, Holzenthal and Blahnik 2004: 14 [checklist] — Dumas et al. 2009: 371 [checklist].

*Barypenthus rufipes*: Burmeister 1839: 929 [Type locality: Brazil, Rio de Janeiro, Nova Friburgo; holotype depository: MLUH — type destroyed; male] — Ulmer 1905a: 20 [male] — Paprocki and Holzenthal 2002: 225 [to synonymy].

*Musarna aperiens*: Walker 1860: 178 [Type locality: South America; holotype depository: BMNH; female] — Ulmer 1905a: 23 [to synonymy] — Betten and Mosely 1940: 222 [type female; redescription] — Paprocki and Holzenthal 2002: 225 [to synonymy].

*Musarna interclusus:* Walker 1860: 178 [Type locality: Brazil; holotype depository: BMNH; female] — Betten and Mosely 1940: 222 [female; redescription] — Paprocki and Holzenthal 2002: 225 [to synonymy].

*Musarna claudens*: Walker 1860: 179 [Type locality: Brazil; holotype depository: BMNH; male] — Betten and Mosely 1940: 222 [male; redescription] — Flint 1969: 24 [larva; pupa; distribution] — Paprocki and Holzenthal 2002: 225 [to synonymy].

*Barypenthus ferrugineus*: Navás 1934: 171 [Type locality: Brazil, Rio de Janeiro, Barão Homem de Melo [currently Itatiaia municipality]; holotype depository: DEIC; male] — Paprocki and Holzenthal 2002: 225 [to synonymy].

*Barypenthus chysopus*: Navás 1934: 172 [Type locality: Brazil, Rio de Janeiro, Barão Homem de Melo [currently Itatiaia municipality]; holotype depository: DEIC; male] — Paprocki and Holzenthal 2002: 225 [to synonymy].

**Sites in Itatiaia massif:** CB 11, CB 14, CB 15, CB 17, CB 18, CB 21, PR 03, PR 12, and PR 14.

Distribution: Brazil (MG, RJ, SP).

#### 61. *Marilia aiuruoca* Dumas and Nessimian, 2009

*Marilia aiuruoca:* Dumas and Nessimian 2009: 344 [Type locality: Brazil, Minas Gerais, Itamonte, Rio Aiuruoca, 22°20′9.28″S 44°41′6.06″W, 1860 m; holotype depository: DZRJ; male; female].

**Sites in Itatiaia massif:** CB 03, CB 19, AI 04, AI 06, PR 09, PR 12, and PR 14.

Distribution: Brazil (MG, RJ).

#### 62. *Marilia huamantincoae* Dumas and Nessimian, 2009

*Marilia huamantincoae*: Dumas and Nessimian 2009: 345 [Type locality: Brazil, Rio de Janeiro, Itatiaia, Maromba, Escorrega do Maromba, Rio Preto, 22°19′48.81″S 44°36′53.94″W, 1357 m; holotype depository: DZRJ; male; female].

**Sites in Itatiaia massif:** PE 01, PE 04, and PR 13.

Distribution: Brazil (RJ).

#### 63. *Marilia major* Müller, 1880

*Marilia major*: Müller 1880: 127 [Type locality: Brazil, Santa Catarina; holotype depository: unknown; case] — Ulmer 1905a: 25 [male] — Blahnik, Paprocki and Holzenthal: 5 [distribution] — Paprocki, Holzenthal and Blahnik 2004: 13 [checklist] — Dumas and Nessimian 2009: 347 [female; distribution].

**Sites in Itatiaia massif:** CB 02, AI 04, PR 06, PR 09, PR 10, and PR 12.

Distribution: Brazil (MG, PR, RJ, SC).

### PHILOPOTAMIDAE

#### 64. *Alterosa beckeri* Blahnik, 2005

*Alterosa beckeri*: Blahnik 2005: 14 [Type locality: Brazil, Rio de Janeiro, Itatiaia, 2100 m; holotype depository: MZSP; male; in *sanctipauli* group] — Dumas et al. 2009: 361 [checklist] — Dumas et al. 2010: 8 [distribution].

**Sites in Itatiaia massif:** AI 06, CB 20, and PR 12.

Distribution: Brazil (MG, RJ).

#### 65. *Alterosa escova* Blahnik, 2005

*Alterosa escova*: Blahnik 2005: 21 [Type locality: Brazil, São Paulo, small stream on São Paulo Route 247, 11 km SE Bananal, 22°45.684′S, 44°23.190′W, 675 m; holotype depository: MZSP; male; in *marinonii* group] —Dumas et al. 2009: 361 [checklist].

**Sites in Itatiaia massif:** CB 11.

Distribution: Brazil (RJ, SP).

#### 66. *Alterosa falcata* Blahnik, 2005

*Alterosa falcata*: Blahnik 2005: 22 [Type locality: Brazil, Minas Gerais, Ibitipoca, sitio of Anestis Papadopoulos, cachoeira, 21°43.441′S 43°54.537′W, 1125 m; holotype depository: MZSP; male; in *falcata* group] — Dumas et al. 2009: 361 [checklist].

**Sites in Itatiaia massif:** CB 15, CB 21, PE 01, PE 04, PR 12, PR 14, and SA 05.

Distribution: Brazil (MG, RJ, SP).

#### 67. *Alterosa flinti* Blahnik, 2005

*Alterosa flinti*: Blahnik 2005: 26 [Type locality: Brazil, Espírito Santo, 24 km SE Santa Teresa, 280 m; holotype depository: MZSP; male; in *marinonii* group] — Dumas et al. 2009: 361 [checklist].

**Sites in Itatiaia massif:** PE 01, PE 02, and PE 04.

Distribution: Brazil (ES, RJ).

#### 68. *Alterosa itatiaiae* Blahnik, 2005

*Alterosa itatiaiae*: Blahnik 2005: 35 [Type locality: Brazil, Rio de Janeiro, Parque Nacional Itatiaia, Rio Campo Belo, trail to Veu da Noiva, 22°25.706′S 44°37.171′W, 1310 m; holotype depository: MZSP; male; in *sanctipauli* group] — Dumas et al. 2009: 362 [checklist].

**Sites in Itatiaia massif:** CB 07, CB 08, CB 11, CB 12, CB 20, CB 21, and PE 01.

Distribution: Brazil (RJ).

#### 69. *Alterosa truncata* Blahnik, 2005

*Alterosa truncata*: Blahnik 2005: 21 [Type locality: Brazil, Minas Gerais, [São Gonçalo do Rio Abaixo municipality], Estação Ecológica de Peti, Córrego Brucutu, 19°52.995′S 43°22.452′W; holotype depository: MZSP; male; in *sanctipauli* group] — Dumas et al. 2009: 362 [checklist; distribution].

**Sites in Itatiaia massif:** SA 03.

Distribution: Brazil (MG, RJ, SP).

#### 70. *Chimarra (Curgia) beckeri* Flint, 1998

*Chimarra beckeri*: Flint 1998: 19 [Type locality: Brazil, Rio de Janeiro, Mangaratiba; holotype depository: MZSP; male, in *morio* group] — Paprocki, Holzenthal and Blahnik 2004: 14 [checklist] — Dumas et al. 2009: 362 [checklist] — Dumas et al. 2010: 8 [distribution].

**Sites in Itatiaia massif:** AI 08, CB 07, CB 08, CB 16, PE 01, PE 02, PE 03, and PE 04.

Distribution: Brazil (MG, RJ).

#### 71. *Chimarra (Chimarrita) camella* Blahnik, 1997

*Chimarra camella*: Blahnik 1997: 219 [Type locality: Brazil, Minas Gerais, Serra do Cipó, Km 116; holotype depository: MZSP; male; in *simpliciforma* group] — Blahnik, Paprocki and Holzenthal 2004: 5 [distribution] — Paprocki, Holzenthal and Blahnik 2004: 14 [checklist] — Dumas et al. 2009: 362 [checklist].

**Sites in Itatiaia massif:** CB 07, CB 15, PE 01, and PE 04.

Distribution: Brazil (MG, RJ, SP).

#### 72. *Chimarra (Chimarrita) camura* Blahnik, 1997

*Chimarra camura*: Blahnik 1997: 222 [Type locality: Brazil, Rio de Janeiro, Km 54 26 Km E Nova Friburgo; holotype depository: MZSP; male; female; in *simpliciforma* group] — Blahnik, Paprocki and Holzenthal 2004: 5 [distribution] — Paprocki, Holzenthal and Blahnik 2004: 14 [checklist] — Dumas et al. 2009: 362 [checklist].

**Sites in Itatiaia massif:** CB 07, CB 09, PE 01, PE 02, PE 05, and PR 03.

Distribution: Brazil (RJ, SP).

#### 73. *Chimarra (Curgia) froehlichi* Flint, 1998

*Chimarra froehlichi*: Flint 1998: 16 [Type locality: Brazil, Rio de Janeiro, Km 54, 26 Km E Nova Friburgo; holotype depository: MZSP; male; in *morio* group] — Blahnik, Paprocki and Holzenthal 2004: 5 [distribution] — Paprocki, Holzenthal and Blahnik 2004: 14 [checklist] — Dumas et al. 2009: 363 [checklist].

**Sites in Itatiaia massif:** CB 06, CB 07, CB 12, CB 16, CB 19, CB 20, CB 21, and PR 12.

Distribution: Brazil (ES, MG, RJ, SP).

#### 74. *Chimarra (Chimarrita) kontilos* Blahnik, 1997

*Chimarra kontilos*: Blahnik 1997: 227 [Type locality: Brazil, Espírito Santo, Caixa d'Água, Santa Teresa; holotype depository: MZSP; male; female; in *simpliciforma* group] — Blahnik, Paprocki and Holzenthal 2004: 5 [distribution] — Paprocki, Holzenthal and Blahnik 2004: 14 [checklist] — Dumas et al. 2009: 363 [checklist].

**Sites in Itatiaia massif:** CB 11, CB 12, CB 13, CB 15, CB 20, CB 22, PE 01, and PE 02.

Distribution: Brazil (ES, MG, RJ, SP).

#### 75. *Chimarra (Curgia) morio* Burmeister, 1839

*Chimarra morio*: Burmeister 1839: 911 [Type locality: Brazil; holotype depository: ZIUH — type lost; female; in *Chimarrha] —* Flint 1998: 14 [male; redescription; variation; distribution; in *morio* group] — Paprocki, Holzenthal and Blahnik 2004: 14 [checklist] — Dumas et al. 2009: 363 [checklist].

*Chimarra martinmoselyi*: Botosaneanu 1980: 98 [replacement name for *Chimarra moselyi* Ross, 1956: 50, 71, preoccupied by *Chimarra moselyi* Denning, 1947: 25; Type locality: Argentina [sic, recte Brazil], Rio de Janeiro, Petrópolis; holotype depository: BMNH; male —Flint 1998: 14 [to synonymy].

**Sites in Itatiaia massif:** PE 01, and PE 05.

Distribution: Brazil (BA, PR, RJ, SC, SP).

#### 76. *Chimarra (Otarrha) odonta* Blahnik, 2002

*Chimarra odonta*: Blahnik 2002: 85 [Type locality: Brazil, São Paulo, [Salesópolis municipality], E.B. Boracéia; holotype depository: MZSP; male; female] — Paprocki, Holzenthal and Blahnik 2004: 14 [checklist] — Dumas et al. 2009: 364 [checklist] — Dumas et al. 2010: 8 [distribution].

**Sites in Itatiaia massif:** AI 04, CB 12, CB 20, CB 21, PE 01, PE 04, PR 03, and SA 03.

Distribution: Brazil (MG, RJ, SP).

### POLYCENTROPODIDAE

#### 77. *Cernotina puri* Dumas and Nessimian, 2011

*Cernotina puri*: Dumas and Nessimian 2011: 32 [Type locality: Brazil, Rio de Janeiro, Itatiaia, Penedo, tributary of Rio Palmital, 22°25′40.0″S 44°32′46.0″W, 584 m; holotype depository: DZRJ; male].

**Sites in Itatiaia massif:** PE 05.

Distribution: Brazil (RJ).

#### 78. *Nyctiophylax (Nyctiophylax) neotropicalis* Flint, 1971

*Nyctiophylax neotropicalis*: Flint 1971: 28 [Type locality: Colombia, Cundinamarca, Rio Sumapaz Gorge, E of Melgar; holotype depository: USNM; male] — Flint 1974b: 39 [distribution] — Angrisano 1994: 138 [distribution] — Blahnik, Paprocki and Holzenthal 2004: 5 [distribution] — Paprocki, Holzenthal and Blahnik 2004: 16 [checklist] — Dumas et al. 2009: 360 [checklist] — Dumas et al. 2010: 9 [distribution].

**Sites in Itatiaia massif:** CB 06, PE 04, and PR 14.

Distribution: Argentina, Brazil (AM, MG, PA, PR, RJ), Colombia, Surinam, and Uruguay.

#### 79. *Polycentropus fluminensis* Hamilton and Holzenthal, 2011

*Polycentropus fluminensis*: Hamilton and Holzenthal 2011: 16 [Type locality: Brazil, Rio de Janeiro, Km 17, 18 Km S of Teresópolis, 1180 m; holotype depository: USNM; male].

**Sites in Itatiaia massif:** AI 06, and CB 14.

Distribution: Brazil (MG, RJ).

#### 80. *Polycentropus inusitatus* Hamilton and Holzenthal, 2011

*Polycentropus inusitatus*: Hamilton and Holzenthal 2011: 48 [Type locality: Brazil, Rio de Janeiro [sic, recte Minas Gerais], [Itamonte municipality], Brejo da Lapa; holotype depository: USNM; male].

**Sites in Itatiaia massif:** Minas Gerais, Itamonte, Brejo da Lapa - *see* Hamilton and Holzenthal 2011.

Distribution: Brazil (MG).

#### 81. *Polycentropus itatiaia* Hamilton and Holzenthal, 2011

*Polycentropus itatiaia*: Hamilton and Holzenthal 2011: 30 [Type locality: Brazil, Rio de Janeiro, Itatiaia, Parque Nacional do Itatiaia, trib. to Rio Taquaral, 22°26.688′S 44°36.464′W, 1320 m; holotype depository: MZSP; male].

**Sites in Itatiaia massif:** PE 01.

Distribution: Brazil (MG, RJ).

#### 82. *Polycentropus rosalyae* Hamilton and Holzenthal, 2011

*Polycentropus rosalyae*: Hamilton and Holzenthal 2011: 42 [Type locality: Brazil, São Paulo, Parque Estadual de Campos de Jordão, Rio Galharda, 22°41.662′S 45°27.783′W, 1530 m; holotype depository: MZSP; male].

**Sites in Itatiaia massif:** AI 04, and CB 02.

Distribution: Brazil (MG, RJ).

#### 83. *Polycentropus urubici* Holzenthal and Almeida, 2003

*Polycentropus urubici*: Holzenthal and Almeida 2003: 26 [Type locality: Brazil, Paraná, Telêmaco Borba, Reserva Samuel Klabin, 24°17′S 50°37′W, 750 m; holotype depository: DZUP; male] — Paprocki, Holzenthal and Blahnik 2004: 16 [checklist] — Dumas et al. 2010: 9 [distribution].

**Sites in Itatiaia massif:** AI 04, and AI 08.

Distribution: Brazil (MG, PR, SC).

#### 84. *Polyplectropus alatespinus* Chamorro-Lacayo and Holzenthal, 2010

*Polyplectropus alatespinus*: Chamorro-Lacayo and Holzenthal 2010: 52 [Type locality: Brazil, Minas Gerais, Parque Estadual do Ibitipoca, Córrego dos Macacos, 21°42′33″S 44°53′36″, 1360 m; holotype depository: MZSP; male; female; in *annulicornis* group].

**Sites in Itatiaia massif:** CB 03, and CB 07.

Distribution: Brazil (MG, RJ, SP).

#### 85. *Polyplectropus annulicornis* Ulmer, 1905

*Polyplectropus annulicornis*: Ulmer 1905: 91 [Type locality: Brazil, Rio Grande do Sul; holotype depository: NMW; female] — Flint 1966: 4 [lectotype; male; female] — Chamorro-Lacayo and Holzenthal 2010: 56 [male; female; distribution; in *annulicornis* group].

**Sites in Itatiaia massif:** PE 01, and PE 04.

Distribution: Brazil (PR, RJ, RS, SC).

#### 86. *Polyplectropus brasilensis *Chamorro-Lacayo and Holzenthal, 2010

*Polyplectropus brasilensis*: Chamorro-Lacayo and Holzenthal 2010: 78 [Type locality: Brazil, Rio de Janeiro, Nova Friburgo, municipal water supply, 950 m; holotype depository: MZSP; male; in *bredini* group].

**Sites in Itatiaia massif:** CB 07.

Distribution: Brazil (RJ, SP).

#### 87. *Polyplectropus hystricosus* Chamorro-Lacayo and Holzenthal, 2010

*Polyplectropus hystricosus*: Chamorro-Lacayo and Holzenthal 2010: 60 [Type locality: Brazil, Minas Gerais, Parque Parque Nacional do Caparaó, Rio Caparaó, Vale Verde, 20°25′02″S 41°50′46″W, 1100 m; holotype depository: MZSP; male; in *annulicornis* group].

**Sites in Itatiaia massif:** CB 04, PR 12, and PR 14.

Distribution: Brazil (RJ, MG).

### XIPHOCENTRONIDAE

#### 88. *Xiphocentron (Antillotrichia) steffeni* (Marlier, 1964)

*Xiphocentron steffeni*: Marlier 1964: 6 [Type locality: Brazil, São Paulo, Boraceia; holotype depository: ISBN; male; in *Melanotrichia*] — Schmid 1982: 114 [comb, nov., as *Xiphocentron steffeni*] — Paprocki, Holzenthal and Blahnik 2004: 16 [checklist] — Dumas et al. 2009: 361 [checklist; distribution] —Dumas et al. 2010: 9 [distribution].

**Sites in Itatiaia massif:** AI 04, CB 15, PE 03, and PE 04.

Distribution: Brazil (MG, RJ, SP).

#### Additional records

The species records listed below were kindly provided by Dr. Ralph W. Holzenthal and Dr. Roger J. Blahnik, both of University of Minnesota, Minnesota, USA. The specimens are deposited in the University of Minnesota Insect Collection (UMSP), Minessota, USA.

### HYDROBIOSIDAE

#### 89. *Atopsyche (Atopsaura) acahuana* Schmid, 1989

*Atopsyche acahuana*: Schmid 1989: 117 [Type locality: Brazil, Ed. ES [Espírito Santo], 15 Km SE Santa Teresa, Fazenda Santa Clara; holotype depository: MZSP; male; in *longipennis* group] — Blahnik, Paprocki and Holzenthal 2004: 4 [distribution] — Paprocki, Holzenthal and Blahnik 2004: 7 [checklist] — Dumas et al. 2009: 364 [checklist].

**Sites in Itatiaia massif:** Rio de Janeiro, Itatiaia municipality, PNI, Rio Campo Belo (22°27′02″S 44°36′49″W), 1300 m — UMSP. Distribution: Brazil (ES, RJ).

### HYDROPSYCHIDAE

#### 90. *Smicridea (Rhyacophylax) radula* Flint, 1974

*Smicridea radula*: Flint 1974c: 36 [Type locality: Costa Rica, San José, Río General, Pacuse; holotype depository: NMNH; male; female].

**Sites in Itatiaia massif:** Rio de Janeiro, Itatiaia municipality, PNI, Rio Campo Belo (22°27′02″S 44°36′49″W), 1300 m — UMSP. Distribution: Brazil (RJ), Costa Rica, El Salvador, Guatemala, Honduras, Mexico, and Panama.

#### 91. *Smicridea (Rhyacophylax) iguazu* Flint, 1983

*Smicridea iguazu*: Flint 1983: 60 [Type locality: Argentina, Pcia. Misiones, Río Iguazú, Camp Nañdu; holotype depository: NMNH; male] — Marinoni and Almeida 2000: 286 [distribution] — Paprocki, Holzenthal and Blahnik 2004: 9 [checklist] — Dumas et al. 2009: 359 [checklist].

**Sites in Itatiaia massif:** Rio de Janeiro, Itatiaia municipality, PNI, Rio Taquaral (22°27′15″S 44°36′34″W), 1300 m; PNI, Rio Campo Belo (22°27′02″S 44°36′49″W), 1300 m — UMSP.

Distribution: Argentina, and Brazil (MG, PR, RJ, SC).

### LEPTOCERIDAE

#### 92. *Nectopsyche pantosticta* Flint, 1983

*Nectopsyche pantosticta*: Flint 1983: 71 [Type locality: Argentina, Pcia. Misiones, Arroyo Coatí, 15 Km E San José; holotype depository: NMNH; male] — Blahnik, Paprocki and Holzenthal 2004: 5 [distribution] — Paprocki, Holzenthal and Blahnik 2004: 13 [checklist] — Dumas et al. 2009: 369 [checklist].

**Sites in Itatiaia massif:** Rio de Janeiro, Itatiaia municipality, PNI, Rio Campo Belo (22°27′02″S 44°36′49″W), 1300 m; PNI, Rio Campo Belo, trail to Véu da Noiva (22°25′42″S 44°37′10″W), 1310 m; PNI, tributary to Rio Taquaral (22°26′41″S 44°36′28″W), 1320m—UMSP.

Distribution: Argentina, and Brazil (RJ, RS).

## Discussion

Itatiaia massif caddisfly fauna represents a significant proportion of Brazilian known fauna. Considering that Brazil has approximately 550 described species (Santos et al. 2011), the fauna of Itatiaia massif comprises about 17% of the total number of species known from the country. This species richness is even more significant considering the minute surface area of Itatiaia massif and the total number of species found in comparison with other South American countries (Argentina, 250 spp.; Venezuela, 240 spp.; Peru, 224 spp.; Chile, 214 spp.; Colombia, 205 spp.; Surinam, 124 spp.) (Angrisano 1995b; Flint 1974b; Flint 1996; Johanson and Holzenthal 2004; Muñoz-Quesada 2000; Rojas 2006). However, it is important to emphasize that several of these countries have deficient knowledge of caddisflies fauna, with most biomes poorly sampled.

Such high species richness present in Itatiaia massif may be explained by the singular features of Mantiqueira mountain range. The mountaintops of Mantiqueira present a cold, temperate climate within a tropical zone and a temperate vegetation island surrounded by a tropical rain forest. Besides that, rocky outcrops abruptly raised from surrounding plains (inselbergs) have a strong influence on the distribution and abundance of biodiversity worldwide, being biological hotspots and supporting unique biotic communities. Moreover, these areas are characterized by high levels of endemism (Porembski et al. 1997; Porembski and Barthlott 2000). This may be attributed to three main factors that have acted at different time scales: biotic evolution in response to climatic and geological history; species adaptations to environmental constraints; and biotic exchanges with the surrounding lowlands (Sarmiento 2002).

Furthermore, 13 species are endemic to Itatiaia massif. This notable mark may reflect the fact that most distributional records of Neotropical caddisflies species are represented by incidental collections, with many species known only from the original site where they were described (Blahnik et al. 2004). However, some of these endemisms are remarkable.

A few conclusive empirical works have focused on the Southern Hemisphere biogeographical patterns (Sanmartín and Ronquist 2004; Giribet and Edgecombe 2006; Daugeron et al. 2009). Two climatic biotic provinces are recognized within Gondwana: the northern Tropical Gondwana, which includes northern South America, Africa, Madagascar, India, New Guinea, and northern Australia; and the southern Temperate Gondwana, which includes southern South America, South Africa, Australia, Antarctica, New Caledonia, and New Zealand (Sanmartín and Ronquist 2004). In the Neotropical Trichoptera, some of these affinities are discussed by de Moor and Ivanov (2008) and Holzenthal and Blahnik (2010).

Some interesting patterns of species distribution occur in Itatiaia massif and other areas of Mantiqueira mountain range. Some endemic species encountered do not seem to be related to any other species in South America, being ancient relicts of prerupture Gondwanan fauna (Flint 1976). The genus *Neoatriplectides* Holzenthal, 1997 contains two species, one in the Mantiqueira mountain range — *N*. *desiderata* Dumas and Nessimian, 2009 — and other in tropical Andes — *N*. *froehlichi* Holzenthal, 1997. The other genera of Atriplectididae, *Atriplectides* Mosely, 1936 and *Hughscottiella* Ulmer, 1910, possess representatives in Australia and the Seychelles Islands, respectively (Holzenthal 1997). Therefore, the family Atriplectididae contains Tropical Gondwana components. Likewise, the monobasic genera *Neoathripsodes* Holzenthal, 1989 (Leptoceridae) and *Barypenthus* Burmeister, 1839 (Odontoceridae) show affinities with South African genera (Holzenthal 1989; de Moor 1997). In contrast, Holzenthal and Blahnik (2010) recently described *Notidobiella brasiliana* Holzenthal and Blahnik, 2010 from Parque Estadual de Campos de Jordão, a neighboring area of the Itatiaia massif at the Mantiqueira mountain range. The genus *Notidobiella* Schmid, 1955 (Sericostomatidae) has three species previously described for southern Chile, what may lead to think in a Temperate Gondwana pattern of distribution. However, the presence of two additional recently described species from Brazilian Amazon basin and Ecuador — *N*. *amazoniana* Holzenthal and Blahnik, 2010 and *N.* *ecuadorensis* Holzenthal and Blahnik, 2010, respectively — may represent an older occurrence in the region, followed by recent dispersal from southern South America to north and its subsequent diversification (Holzenthal and Blahnik 2010).

Some other curious distributional patterns can be observed in *Antarctoecia brasiliensis* Huamantinco and Nessimian, 2003, the single species of Limnephilidae recorded from Brazil. The genus *Antarctoecia* Ulmer, 1907 shows a disjunct distribution, with *A. nordenskioeldii* (Ulmer, 1907), described from the Puna de Jujuy (Argentina) in elevations above 4,500 m, and *A*. *brasiliensis*, found only at Itatiaia plateau above 1,800 m. Biogeographical affinities have been recognized between the fauna and flora of southeastern Brazilian mountains and the Andean-Patagonian region (Iilies 1969; Sick 1985; Safford 1999; Behling 2002). It is assumed that Brazilian southeastern mountains were colonized by Andean elements during Pleistocene glaciations, when climatic-vegetational connections between both regions were similar (Simpson-Vuilleumier 1971). During interglacial periods, those montane habitats would have retreated to cooler upland areas, explaining the isolated occurrence of some Andean-Patagonian taxa on southeastern Brazilian mountaintops (Simpson 1979; Safford 1999). Holzenthal and Blahnik (2010) claim that the distributional pattern of *Notidobiella* and *Antarctoecia,* like other with Patagonian and Neotropical distribution, are probably one and the same.

de Moor and Ivanov (2008) propose five biogeographical patterns. One of them is a two-way exchange of Neotropic and Neartic faunas. It may be the case of *Anastomoneura guaybae* Huamantinco and Nessimian, 2004 (Odontoceridae), the only species within the genus, which larvae share more similarities with the North American genus *Nerophilus* Banks, 1899 and *Namamyia* Banks, 1905 (Dumas and Nessimian 2006).

Itatiaia massif is located in the Atlantic Forest biome, which is classified as one of the 25 biodiversity hotspots around the world. However, less than 8% of the original forest of this biome now remains, and it occurs mostly in isolated remnants scattered throughout a landscape dominated by agricultural uses. Despite these disturbances, Atlantic Forest is still extremely rich in biodiversity, sheltering a significant proportion of total national fauna and flora, with high levels of endemism (Dean 1997; Joly and Bicudo 1998; Myers et al. 2000). Although part of Itatiaia massif is inserted in protected areas — Parque Nacional do Itatiaia and Área de Proteção Ambiental da Mantiqueira — there are constant losses of its vegetation and fauna by anthropic pressures, such as fire and livestock grazing.

The results of this study, allied with other faunistic and floristic works (Geise et al. 2004; Sendas and Araújo 2004; Ribeiro et al. 2007; Monné et al. 2009; among others), emphasize the importance of better efforts to preservation and conservation of Itatiaia massif. Furthermore, considering the degree of threat in the Atlantic Forest in this area and its high biological diversity, including that of Trichoptera, the conservation of these remnants and incentives to continue studying native species of fauna and flora are therefore highly recommended. The creation of new conservation units, or the enlargement of existing ones, is necessary to better preserve the biodiversity and to help prevent further deforestation.

## Editor's note

Paper copies of this article will be deposited in the following libraries. Universitaetsbibliothek Johann Christian Senckenberg, Frankfurt Germany; National Museum of Natural History, Paris, France; Field Museum of Natural History, Chicago, Illinois, USA; University of Wisconsin, Madison, USA; University of Arizona, Tucson, Arizona, USA, Smithsonian Institution Libraries, Washington, D.C., USA; The Linnean Society, London, England. The date of publication is given in ‘About the Journal’ on the JIS website.

**Table 1.**
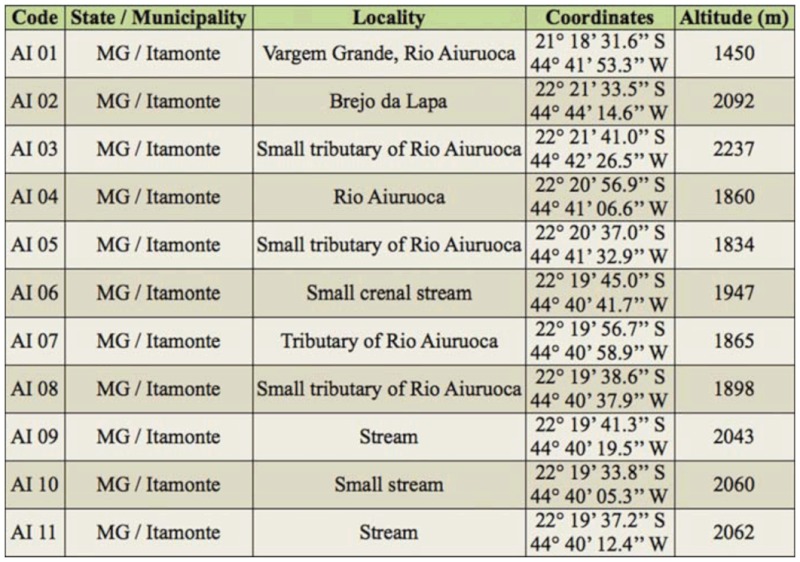
Sampling sites in Rio Aiuruoca drainage sub-basin.

**Table 2.**
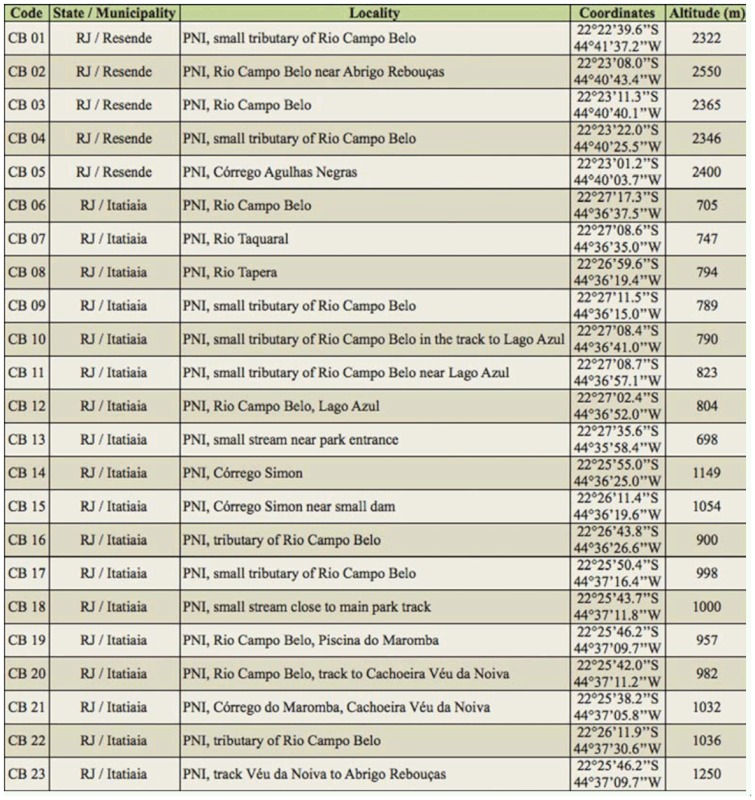
Sampling sites in Rio Campo Belo drainage sub-basin.

**Table 3.**
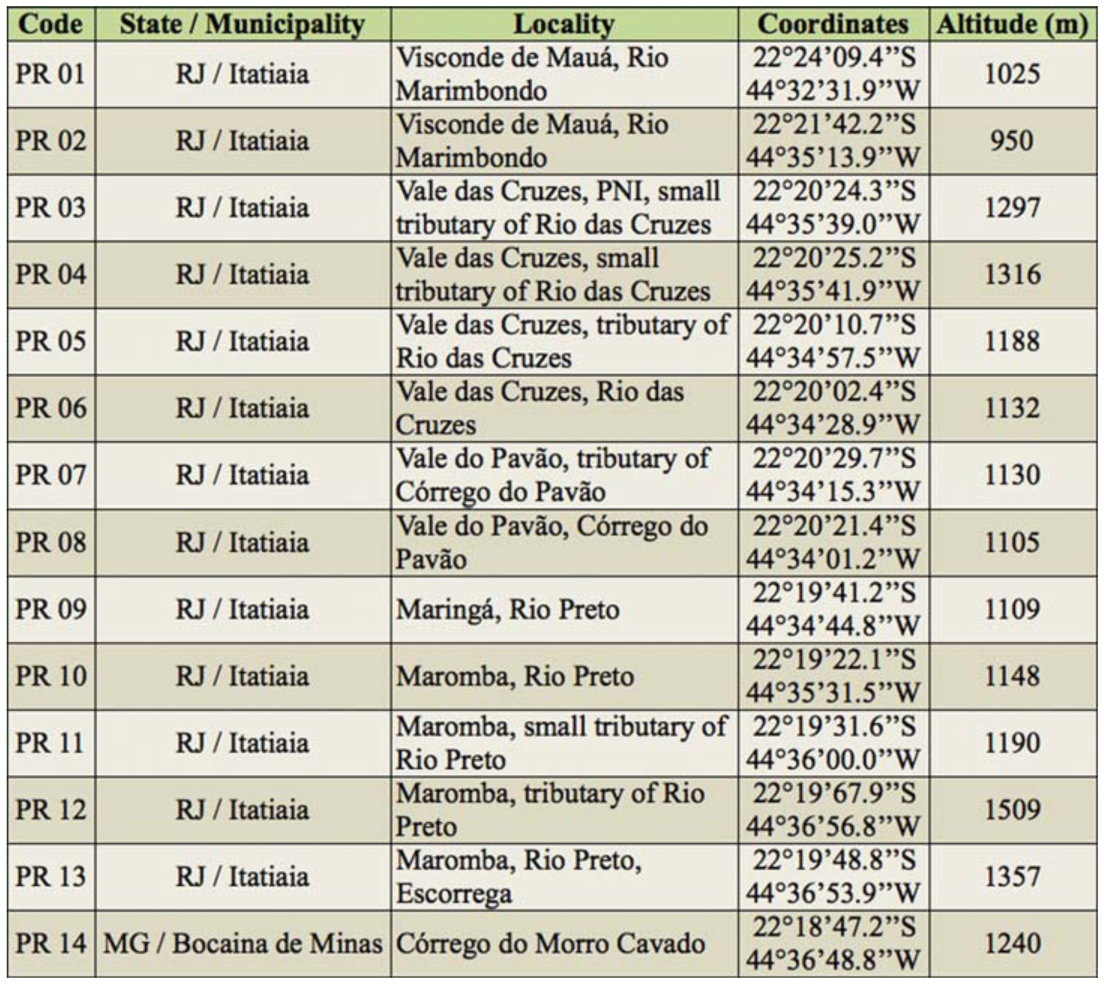
Sampling sites in Rio Preto drainage sub-basin.

**Table 4.**
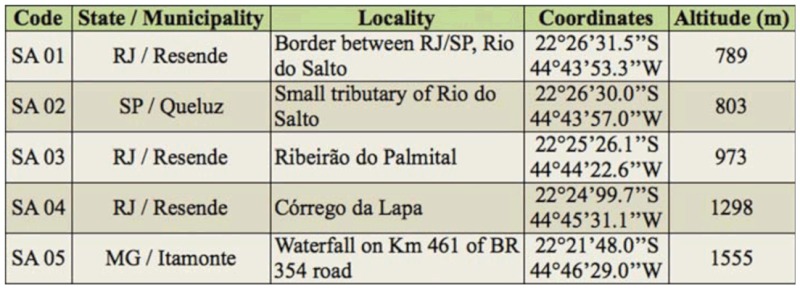
Sampling sites in Rio do Salto drainage sub-basin.

**Table 5.**
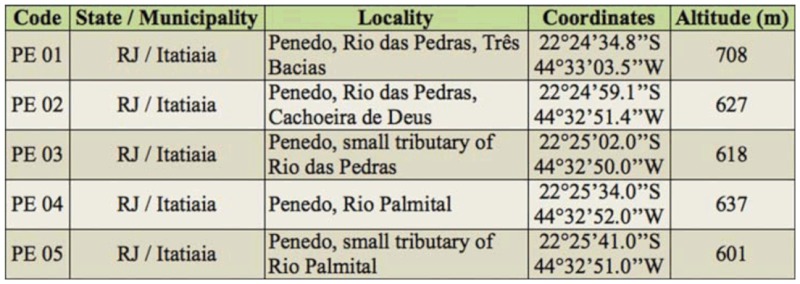
Sampling sites in Rio das Pedras drainage sub-basin.
